# Modulation of Peripheral CD4^+^CD25^+^Foxp3^+^ Regulatory T Cells Ameliorates Surgical Stress-Induced Atherosclerotic Plaque Progression in ApoE-Deficient Mice

**DOI:** 10.3389/fcvm.2021.682458

**Published:** 2021-08-12

**Authors:** Jessica Handke, Laura Kummer, Markus A. Weigand, Jan Larmann

**Affiliations:** Department of Anesthesiology, Heidelberg University Hospital, Heidelberg, Germany

**Keywords:** atherosclerosis, regulatory T cells, inflammation, perioperative stress response, plaque vulnerability

## Abstract

Systemic inflammation associated with major surgery rapidly accelerates atherosclerotic plaque progression in mice. Regulatory T cells (Tregs) have emerged as important modulators of atherogenesis. In coronary artery disease patients, low frequency of Tregs constitutes an independent risk factor for cardiovascular complications after non-cardiac surgery. In this exploratory analysis, we investigate whether preoperative Treg levels affect surgery-induced atherosclerotic lesion destabilization in a murine model of perioperative stress. After 9 weeks of high-cholesterol diet, atherosclerotic apolipoprotein E-deficient mice with modulated Treg levels were subjected to a 30-minute surgical procedure consisting of general isoflurane anesthesia, laparotomy and moderate blood loss. Controls underwent general anesthesia only. Brachiocephalic arteries were harvested 3 days after the intervention for histomorphological analyses of atherosclerotic plaques. Tregs were depleted by a single dose of anti-CD25 monoclonal antibody (mAb) administered 6 days prior to the intervention. Expansion of Tregs was induced by daily injections of IL-2/anti-IL-2 complex (IL-2C) on three consecutive days starting 3 days before surgery. Isotype-matched antibodies and PBS served as controls. Antibody-mediated modulation was Treg-specific. IL-2C treatment resulted in an eight-fold elevation of peripheral CD4^+^CD25^+^Foxp3^+^ Tregs compared to mice administered with anti-CD25 mAb. In mice treated with PBS and anti-CD25 mAb, surgical stress response caused a significant increase of atherosclerotic plaque necrosis (PBS: *p* < 0.001; anti-CD25 mAb: *p* = 0.037). Preoperative Treg expansion abrogated perioperative necrotic core formation (*p* = 0.556) and significantly enhanced postoperative atherosclerotic plaque stability compared to PBS-treated mice (*p* = 0.036). Postoperative plaque volume (*p* = 0.960), stenosis (*p* = 0.693), lesional collagen (*p* = 0.258), as well as the relative macrophage (*p* = 0.625) and smooth muscle cell content (*p* = 0.178) remained largely unaffected by preoperative Treg levels. In atherosclerotic mice, therapeutic expansion of Tregs prior to major surgery mitigates rapid effects on perioperative stress-driven atherosclerotic plaque destabilization. Future studies will show, whether short-term interventions modulating perioperative inflammation qualify for prevention of cardiovascular events associated with major non-cardiac surgery.

## Introduction

Annually, clinical manifestations of atherosclerosis, such as ischemic heart disease and stroke, account for 15 million deaths, globally (1). Although atherogenesis is generally regarded insidious in nature, numerous studies also describe more rapid, accelerated lesion progression triggered by physical and inflammatory stressors (2–5). In line with these findings, we and others have demonstrated that systemic inflammation associated with major surgical interventions rapidly accelerates atherosclerotic plaque progression in a murine model of perioperative stress (6, 7).

Acute myocardial infarction (MI) is seen in up to 5.8% of cardiovascular risk patients undergoing major non-cardiac surgery and has been independently associated with increased perioperative mortality (8–10). Coronary plaque rupture accounts for 13–59% of perioperative MIs (11–13). Factors triggering perioperative plaque rupture include endocrinological dysregulation, inflammation, hypercoagulability, and increased hemodynamic strain in response to surgical trauma and concomitant medication (14, 15). Elucidating the pathophysiological mechanisms underlying perioperative MI will aid to develop effective preventive measures and treatment options.

Low-grade, chronic inflammatory response mediated by cells of the innate and adaptive immune system significantly drives atherosclerotic plaque development and progression (16). Immunoregulatory T cell populations exert atheroprotective effects in all stages of atherosclerosis mainly by resolving vascular inflammation (17). CD4^+^CD25^+^Foxp3^+^ regulatory T cells (Tregs) are crucial for maintaining peripheral tolerance to self-antigens and prevention of unrestricted effector T cell expansion (18). Long-term depletion of Tregs in mice increases atherosclerotic plaque development (19, 20) while adoptive transfer of Tregs promotes lesion stability in a dose-dependent manner (21, 22). In patients, low frequency of Tregs is associated with atherosclerotic plaque vulnerability (23) and impaired thymic output of Tregs correlates with increased cardiovascular risk (24, 25). We recently demonstrated, that coronary artery disease patients with low preoperative Treg counts face a higher risk of major adverse cardiovascular and cerebrovascular events after non-cardiac surgery (26). We further provided evidence that increased cardiovascular risk associated to sequential surgical procedures may be due to reduced levels of intraplaque Tregs (27). Here, we explore whether preoperatively low Treg counts further accelerate perioperative destabilization of atherosclerotic plaques and whether preoperative Treg expansion has the potential to limit surgical stress-induced atherosclerosis progression.

## Materials and Methods

### Mice

All experiments were approved by local authorities (Regierungspräsidium Karlsruhe, date of approval 01/03/2018) and were conducted in accordance with national legislation. Animals were maintained under controlled, pathogen-free conditions (22 ± 2°C, 50–60% humidity, 12 h light/dark cycles), fed *ad libitum* and were handled according to the Society of Laboratory Animal Science recommendations. In total, 131 apolipoprotein E-deficient (ApoE^−/−^; 62 male, 69 female) mice, on a C57BL/6 background and bred in-house (Interfacultary Biomedical Faculty, University of Heidelberg, Heidelberg, Germany), were used in this study. Genotyping was performed by Transnetyx (Cordova, TN, US). Starting at 8 weeks of age, mice were set on a high-cholesterol Western diet (WD) containing 1.25% cholesterol (Altromin, Lage, Germany). WD was maintained for the duration of the experiment, such that at the time of sacrifice, mice had been on a WD for 9.5 weeks.

### *In vivo* Regulatory T Cell Modulation

To determine the most suitable time point ensuring maximum Treg modulation at the day of surgery and thereafter, Tregs were quantified at three different time points after initiating antibody treatment. Thus, in a preliminary experiment, mice were randomly assigned to one of the five treatment groups: anti-CD25, IgG1, IL-2/anti-IL-2 complex, IL-2/IgG2, and PBS as control (all from BioLegend, San Diego, CA, US). Treg reduction was initiated by a single intraperitoneal (i.p.) injection of 250 μg anti-CD25 mAb (clone PC61, #102040); an isotype-matched IgG1 (clone G0114F7, #401916) was used as control. IL-2/anti-IL-2 (IL-2C), IL-2/IgG2 and PBS were i.p. administered daily on three consecutive days. IL-2 complexes were formed of 1 μg recombinant IL-2 and 5 μg anti-IL-2 mAb (clone JES-1A12, #503706) or IgG2 (clone RTK2758, #400544), respectively, and incubated for 30 min at 37°C in PBS (28). Blood was collected from the facial vein 6, 8, and 10 days after anti-CD25 mAb and IgG1 administration, and at day 3, 4, and 5 after the first injection of IL-2C, IL-2/IgG2, and PBS, respectively. In the final model, based on the kinetic of Treg modulation, anti-CD25-mediated Treg decrease was induced 6 days prior to surgery whereas IL-2C-mediated Treg expansion was initiated 3 days preoperatively. Controls were treated accordingly.

### Perioperative Stress Model

After 9 weeks of WD, mice were subjected to a perioperative stress model as previously described (6). Briefly, mice were anesthetized using isoflurane inhalation followed by longitudinal laparotomy (approx. length of 1.5 cm) and 400 μl blood withdrawal from the facial vein, which corresponds to an intraoperative blood loss of ~20%. After 30 min, the abdomen was closed using single-knot sutures. Controls underwent 30-minute general anesthesia (sham). To investigate the effect of surgery on Treg counts, sham mice additionally received a 100 μl blood draw from the facial vein during anesthesia. Three days postoperatively, mice were euthanized, exsanguinated, and perfused through cardiac puncture using 0.9% saline at physiological pressure. Brachiocephalic arteries were harvested, embedded in OCT medium and stored at −80°C until further processed for histologic analyses.

### Histological Analyses of Brachiocephalic Atherosclerotic Plaques

Serial cross-sections were prepared at 5 μm thickness through the entire length of the brachiocephalic artery using a cryomicrotome (Leica Microsystems, Wetzlar, Germany). For the detection of Foxp3^+^ Tregs in atherosclerotic plaques, every 15th paraformaldehyde-fixed tissue section was treated with citrate buffer for antigen retrieval. After cell permeabilization in 0.3% Triton X-100 solution, sections were blocked in 2.5% normal goat serum (Vector Laboratories, #MP-5444-15) followed by overnight incubation with the primary rat anti-mouse Foxp3 antibody (1:100; Thermo Fisher Scientific, #14-5773). Secondary staining was performed using the ImmPRESS®-AP anti-rat IgG polymer detection kit (Vector Laboratories, #MP-5444-15) following the manufacturer's instructions. Sections were subsequently treated with hematoxylin solution (Carl Roth, Karlsruhe, Germany) to counterstain nuclei. Thymic tissue was used as positive control. The number of Foxp3^+^ cells was counted manually and normalized to plaque area.

Every 15th section was stained with hematoxylin and eosin (HE) for quantification of atherosclerotic lesion volume and average stenosis. For the analysis of plaque morphology, mice with a maximum stenosis below 10% were excluded, as these were considered preliminary foam cell accumulations not allowing to reliably evaluate lesion stability. For this exploratory analysis, adjacent sections were stained for collagen and necrosis, for macrophages or smooth muscle cells (SMC).

Collagen content and necrotic core (NC) area were quantified on four cross-sections showing the maximum stenosis and stained with Masson's trichrome (Carl Roth, Karlsruhe, Germany) according to the manufacturer's instructions. Collagen was defined as the area that had stained positive for lightgreen above a set threshold and was expressed relative to whole plaque size. Necrosis was defined as anuclear lesion area with total or almost complete loss of collagen and was expressed as mean necrotic area per plaque.

Immunostaining of total CD68-positive macrophages and M2 macrophages was performed as described elsewhere (29). In brief, after cross-sections were fixed in ice-cold acetone, M2 macrophages were identified by dual staining of CD68 (1:300; #MCA1957GA, Bio-Rad, Hercules, CA, USA) and CD206 (1:100; #ab64693, Abcam, Cambridge, UK) for 3 h at room temperature. Sections were subsequently incubated with anti-rabbit IgG Alexa Fluor-555 (#4413S) and anti-rat IgG Alexa Fluor-488 (#4416S), both from Cell Signaling Technology (Danvers, MA, US) and used at dilutions of 1:1,000. Slides were mounted and cover-slipped using fluorescence mounting medium (Dako North America, Carpinteria, CA, US). The number of total macrophages and M2 macrophages was calculated per plaque area.

For fluorescent staining of SMCs an antibody against α smooth muscle actin (1:400; #A2547, Merck, Darmstadt, Germany) and a polyclonal goat anti-mouse IgG secondary antibody conjugated to Alexa Fluor 488 (1:100; #A-11017, Thermo Fisher Scientific, Waltham, MA, US) were used. Cryosections were fixed in ice-cold acetone before treated with M.O.M blocking reagent (Vector Laboratories, Burlingame, CA, US) to reduce endogenous mouse Ig staining. DAPI was used for nuclei counterstaining. Buried fibrous caps (FC) were identified as SMC-rich layers within lesions, that were covered by newly formed plaque material (30).

Following the Stary criteria (31), plaque morphology was further evaluated by scoring features, that indicate lesion complexity, such as necrosis, intraplaque hemorrhage, and buried FCs at the site of maximum stenosis. Four cross-sections stained for collagen and α smooth muscle actin were analyzed for the presence of necrotic area and buried FCs, respectively. Likewise, four HE and Masson's trichrome stainings were used to detect red blood cells indicating intraplaque bleeding. The presence of each individual feature was graded one scoring point, so that complicated plaques containing all three features were rated three points, whereas absence of either criterion was assigned zero points (6).

Images were captured on an Olympus BX63 microscope (Olympus Life Science Solutions, Waltham, MA, US) and analyzed using the CellSens (Olympus Life Science Solutions, Waltham, MA, US) and Fiji imaging platform (32) in an observer-blinded fashion.

### Flow Cytometry

To assess the effect of surgery on Tregs as well as the efficacy and specificity of Treg-modulating treatment, Tregs and leukocyte subpopulations were quantified using flow cytometry. EDTA-anticoagulated whole blood or single cell suspensions prepared from lymphoid organs were treated with Fc-block (mouse TruStain FcX™, BioLegend, San Diego, CA, US) to inhibit unspecific binding. Cells were subsequently stained with fixable viability dye (Thermo Fisher Scientific, Waltham, MA, USA) and fluorescently labeled surface antibodies: anti-CD3 FITC, anti-CD4 PerCP-Cy5.5, anti-CD8 APC-Cy7, anti-CD11b PE, anti-CD25 APC, anti-Ly6-C PerCP-Cy5.5, anti-NK1.1 APC-Cy7 (all from BioLegend, San Diego, CA, US), anti-CD19 APC, anti-Ly6-G FITC (both from BD Biosciences, Heidelberg, Germany), and anti-CD45 eFluor® 450 (Thermo Fisher Scientific, Waltham, MA, USA). For Treg identification, intracellular staining of Foxp3 was performed using an anti-Foxp3 mAb (clone 3G3, #A18690) and the Foxp3 transcription buffer staining set (both from Thermo Fisher Scientific, Waltham, MA, USA) according to the manufacturer's instructions. Isotype controls were included for CD25 staining (IgG1, clone RT4530, BioLegend, San Diego, CA, US). Flow cytometric analyses were performed on a BD FACSVerse™ using the FACSuite Software (BD Biosciences, Heidelberg, Germany) and gating strategies for Treg and leukocyte subpopulation identification are depicted in Supplementary Figures 1, 2, respectively.

### Statistical Analysis

Statistical analyses were performed using the GraphPad Prism software. Data were tested for normality using the Kolmogorov-Smirnov test. As part of the data did not follow normal distribution, non-parametric Mann-Whitney U or Wilcoxon signed-rank test were used as appropriate. Kruskal-Wallis test was performed to test for statistical differences between multiple treatment groups. If the Kruskal-Wallis test revealed *p* < 0.05, Dunn's *post-hoc* test was performed for comparison of anti-CD25 mAb and IL-2C to PBS treated mice, respectively. Differences were considered statistically significant, if *p* < 0.05. Outliers identified based on the ROUT method were excluded from the histomorphometric analyses (33). Data are presented as median (interquartile range); boxes mark interquartile ranges and whiskers represent 5th to 95th percentiles. Median and average values are shown as vertical lines and “+,” respectively. Sex-stratified data for each analysis are included in the Supplementary Material to allow the assessment of sex differences. However, as the study was not adequately powered to separately assess gender-specific effects, descriptive data are provided only.

## Results

### Perioperative Stress Response Induces Treg Expansion in Blood and Thymus

**Figure 1:**
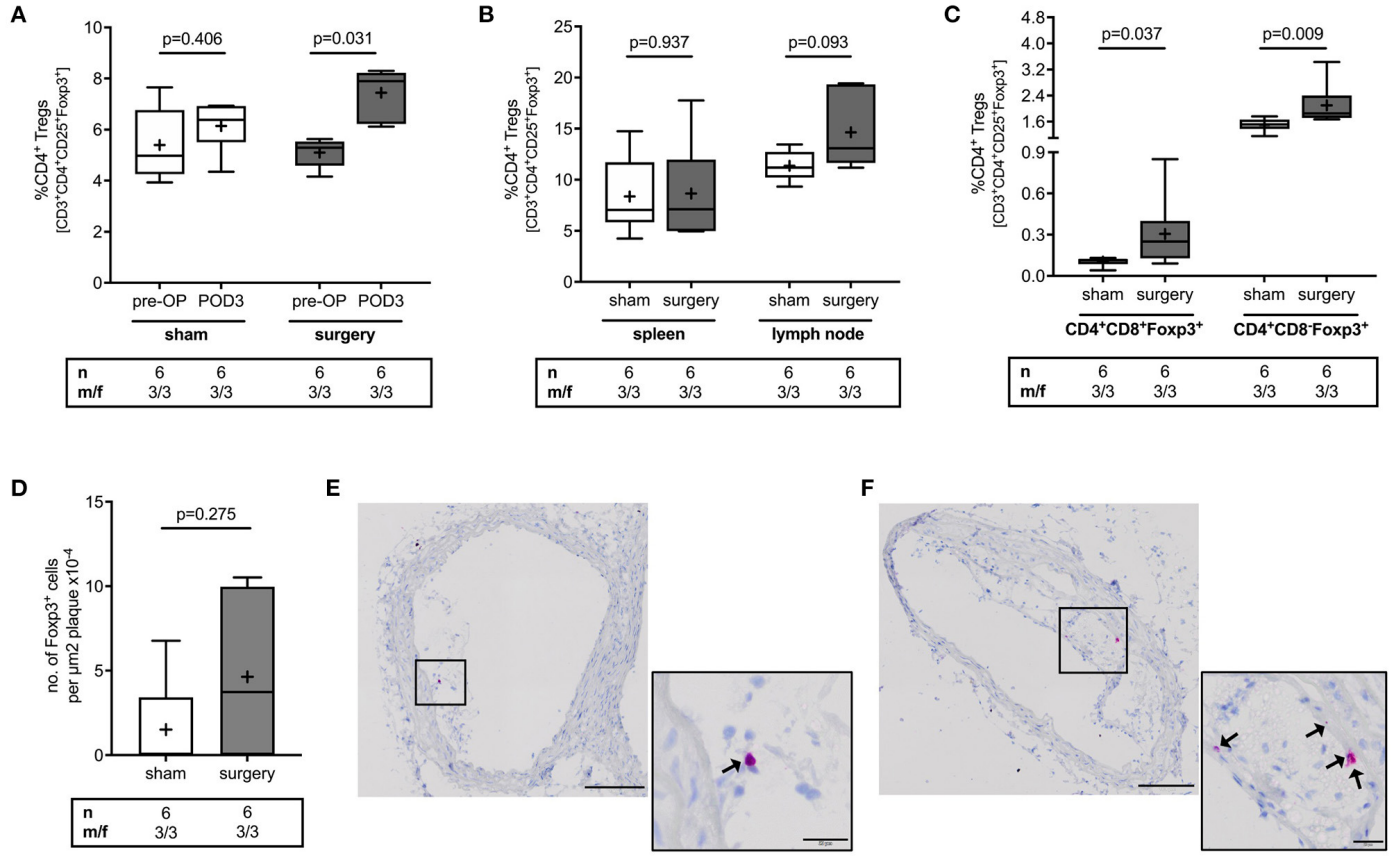
Effect of perioperative stress on regulatory T cell count in blood, spleen, lymph node, thymus and atherosclerotic plaques of ApoE^−/−^ mice. ApoE^−/−^ mice of mixed gender were fed a Western diet for 9 weeks and were exposed to the surgical stress model or the sham intervention at the age of 17 weeks. Sham mice additionally received a 100 μl blood draw at the day of surgery. **(A)** Blood count of Tregs was quantified preoperatively (pre-OP) and on postoperative day 3 (POD3), at the day of sacrifice using flow cytometry. Postoperatively, the number of Tregs was quantified in **(B)** spleen and lymph nodes, **(C)** tyhmus, and **(D–F)** in atherosclerotic plaques of the brachiocepahlic artery. Immunohistochemical images of atherosclerotic plaques showing a representative number of Tregs in mice that underwent **(E)** the sham intervention or **(F)** surgery. Scale bars represent 100 and 20 μm of overview and close-up images, respectively. The total number of mice under study as well as the ratio of male to female mice is indicated per group. Statistical comparisons were performed using non-parametric Wilcoxon signed-rank or Mann-Whitney U test, as appropriate.

To analyze the effect of surgical stress on Treg counts, Tregs were quantified in blood, spleen, lymph nodes, thymus and atherosclerotic plaques in the brachiocephalic artery of mice that either underwent surgery or the sham intervention. Surgery induced a 50% increase in CD4^+^CD25^+^Foxp3^+^ Tregs in blood [5.3 (4.6; 5.5) vs. 7.9 (6.2; 8.2) %CD4^+^ for pre-OP vs. POD3, *p* = 0.031], an effect that was absent in sham mice [5.0 (4.3; 6.8) vs. 6.4 (5.5; 6.9) %CD4^+^ for pre-OP vs. POD3, *p* = 0.406] (Figure 1A). There was no difference in postoperative Treg levels in spleen [7.1 (5.8; 12) vs. 7.1 (5.0; 12) %CD4^+^ for sham vs. surgery, *p* = 0.937] or lymph nodes [11 (10, 13) vs. 13 (12, 19) %CD4^+^ for sham vs. surgery, *p* = 0.093] (Figure 1B). In the thymus, mice with surgery showed higher levels in double (DP) and single positive (SP) Tregs compared to sham animals [DP: 0.12 (0.09; 0.12) vs. 0.25 (0.13; 0.4) %CD4^+^ for sham vs. surgery, *p* = 0.037; SP: 1.5 (1.4; 1.7) vs. 1.9 (1.7; 2.4) %CD4^+^ for sham vs. surgery, *p* = 0.009] (Figure 1C). The number of Tregs within atherosclerotic plaques was 3.7 (0.0; 10) Foxp3^+^ cells per μm^2^ plaque x10^−4^ in mice that underwent surgery vs. 0.0 (0.0; 3.4) in sham animals (*p* = 0.275) (Figures 1D–F). In an exploratory approach, we stratified data for male vs. female. Respective data for Treg alterations in response to surgery for male and female mice are reported in Supplementary Figure 3.

As the rapid expansion of Tregs could be interpreted as a protective effect to counterbalance surgery-induced inflammation, we analyzed whether modulation of preoperative Treg levels may affect inflammation-driven atherosclerotic plaque progression during surgery.

### Antibody-Mediated Treg Modulation

**Figure 2:**
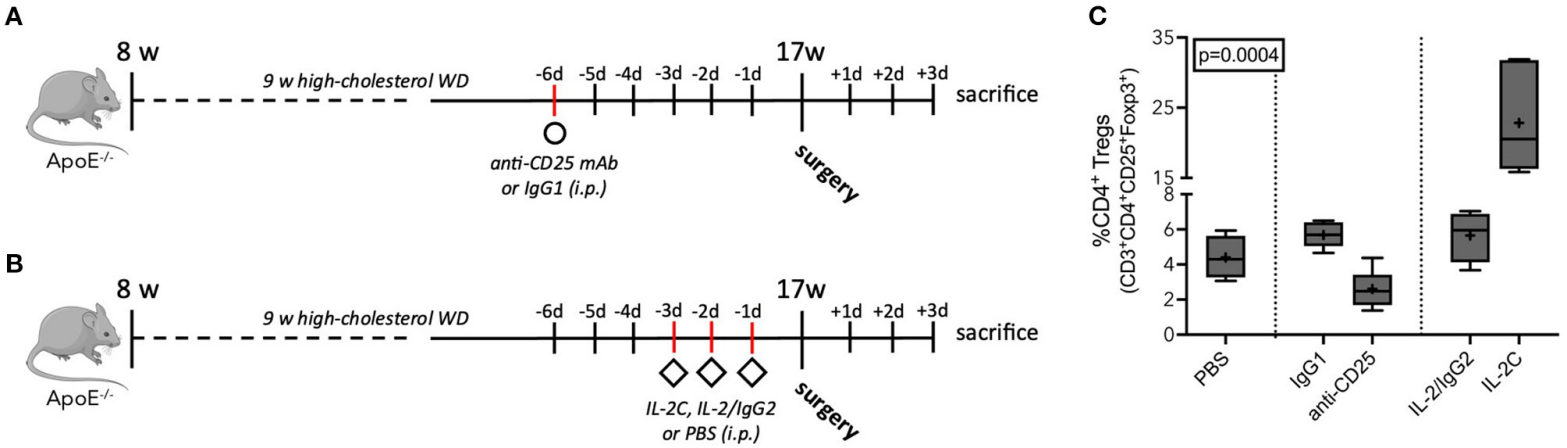
Antibody-mediated Treg modulation in atherosclerotic ApoE^−/−^ mice. **(A)** Schematic of preoperative anti-CD25-mediated Treg downregulation and **(B)** IL-2 complex (IL-2C)-induced expansion of Tregs. ApoE^−/−^ mice of mixed gender fed a Western diet (WD) for 9 weeks, underwent the surgical stress model or sham intervention at the age of 17 weeks, and were sacrificed at postoperative day 3. Treg reduction and expansion was induced by a single injection of anti-CD25 mAb (250 μg per mouse) and three daily injections of IL-2C (complex of 1 μg recombinant IL-2 plus 5 μg anti-IL-2 mAb per mouse), respectively. Isotype-matched antibodies and PBS were used as controls. **(C)** Flow cytometric analysis of circulating CD3^+^CD4^+^CD25^+^Foxp3^+^ Tregs relative to the CD4^+^ T cell population at the day of surgery (*n* = 4–6). Kruskal-Wallis-test was used to assess statistical difference between the five treatment groups.

To ensure maximum Treg modulation at the day of surgery and 3 days thereafter, we determined peripheral Treg levels at three different time points after treatment initiation. Compared to IgG1 controls, a single anti-CD25 mAb administration resulted in 60% decrease of peripheral Treg levels 6 [5.7 (5.0; 6.4) vs. 2.5 (1.7; 3.4) %CD4^+^, *p* = 0.002] and 10 days [7.8 (6.2; 8.7) vs. 3.1 (2.3; 4.6) %CD4^+^, *p* = 0.004] after injection. At 8 days, Tregs were reduced from 4.9 (4.4; 6.0) to 3.2 (2.4; 3.5) %CD4^+^ (*p* = 0.002) (Supplementary Figure 4A). In PBS-treated mice, Tregs accounted for 4.4 (3.3; 5.6) % of the CD4^+^ T cell pool on average. Treg expansion was induced by three daily injections of IL-2C. Treg counts increased to 21 (16, 32) %CD4^+^ (*p* = 0.006) and 21 (11, 29) %CD4^+^ (*p* = 0.002) 3 and 5 days after treatment start, respectively, and reached its peak on day four after treatment initiation [28 (18, 34) %CD4^+^, *p* < 0.001]. Animals treated with IL-2/IgG2 control did not develop increased Treg numbers. The maximum value was seen 4 days after the first injection [6.6 (5.6; 7.5) %CD4^+^, *p* = 0.272] (Supplementary Figure 4B). Based on these results, we implemented the final model with Treg downregulation 6 days before surgery (Figure 2A), while upregulation was induced 3 days preoperatively (Figure 2B). Accordingly, at the day of surgery, IL-2C-treated mice presented with 8-fold elevated peripheral Tregs compared to mice receiving anti-CD25 mAb (Figure 2C) (Kruskal-Wallis *p* = 0.0004). Other peripheral leukocyte subpopulations were not significantly affected by our Treg-modulating treatment (Supplementary Figure 5).

### Preoperative Treg Levels Did Not Affect Postoperative Plaque Volume and Stenosis

To investigate the effect of preoperative Treg levels on perioperative atherosclerotic plaque progression, 17-week-old atherosclerotic ApoE^−/−^ mice with modulated Treg levels underwent a surgical stress model. Morphological characterization of the atherosclerotic lesion phenotype was assessed 3 days post-surgery in cross-sections of the brachiocephalic artery.

**Figure 3:**
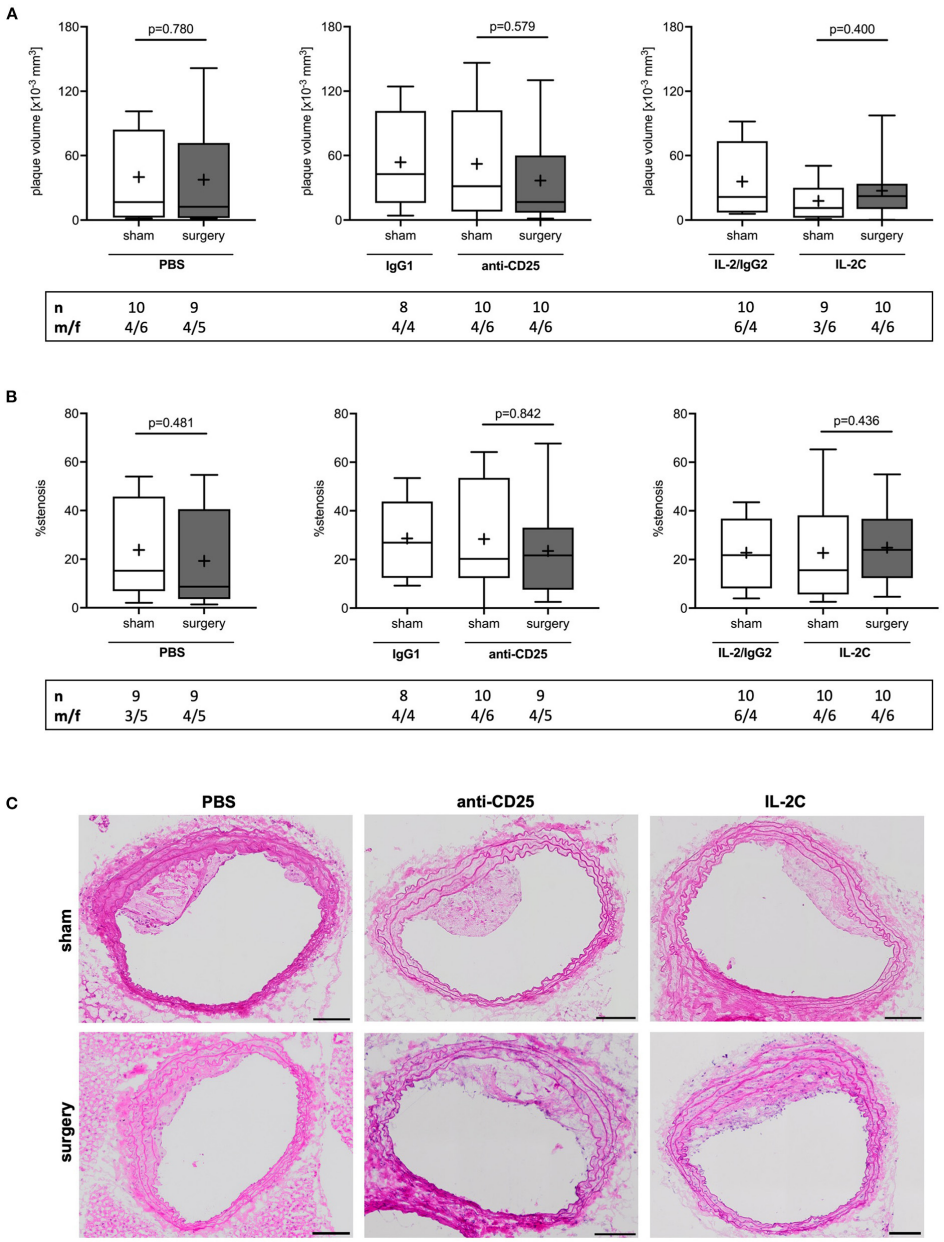
Postoperative plaque volume and stenosis in mice with preoperatively modulated Treg levels. Atherosclerotic ApoE^−/−^ mice with modulated Treg levels were subjected to perioperative stress consisting of 30 min laparotomy combined with moderate blood loss. **(A)** Atherosclerotic plaque volume of the brachiocephalic artery was derived from hematoxylin and eosin-stained sections every 75 μm. **(B)** Mean stenosis was calculated accordingly. **(C)** Representative images of brachiocephalic cross-sections (scale bar 100 μm). The total number of mice under study as well as the ratio of male to female mice is indicated per group. Outliers identified by ROUT were excluded from the analysis resulting in *n* = 8–10 mice per group. Two-tailed Mann-Whitney U was used to compare sham and corresponding intervention groups; Kruskal-Wallis test was used to assess differences between surgical intervention groups (gray; plaque volume *p* = 0.960; % stenosis *p* = 0.693).

Independent of the preoperative Treg level, surgical stress inflicted by laparotomy and moderate blood loss had no effect on perioperative plaque volume growth as compared with sham animals, which received general anesthesia only (PBS: *p* = 0.780; anti-CD25 mAb: *p* = 0.579; IL-2C: *p* = 0.400) (Figure 3A). Moreover, statistical comparison of surgical intervention groups revealed no difference in postoperative plaque volume (Kruskal-Wallis *p* = 0.960). Similar results were obtained for the analysis of mean percentage stenosis (PBS: *p* = 0.481; anti-CD25 mAb: *p* = 0.842; IL-2C: *p* = 0.436) (Figures 3B,C) as well as for plaque area at the site of maximal stenosis (PBS: *p* = 0.905; anti-CD25 mAb: *p* = 0.684; IL-2C: *p* = 0.401) (Supplementary Figure 6A). Likewise, we did neither observe any quantitative difference in the extent of postoperative stenosis (Kruskal-Wallis *p* = 0.693) nor in plaque size at the site of maximum stenosis among surgical intervention groups (Kruskal-Wallis *p* = 0.439). These observations were comparable between male and female mice under study. However, overall plaque size was larger in male compared to female mice (Supplementary Figures 6B,C, 7).

To further assess the effect of preoperative Treg levels on perioperative atherosclerotic lesion progression, multiple markers of plaque instability were analyzed using histochemical stainings. In total, 12 mice had to be excluded from the analysis (PBS: *n* = 6; anti-CD25 mAb: *n* = 4; IL-2C: *n* = 2) as the maximum stenosis was below 10%, thereby not allowing a reliable evaluation of plaque morphology.

### Preoperative Treg Expansion Attenuates Perioperative NC Formation

**Figure 4:**
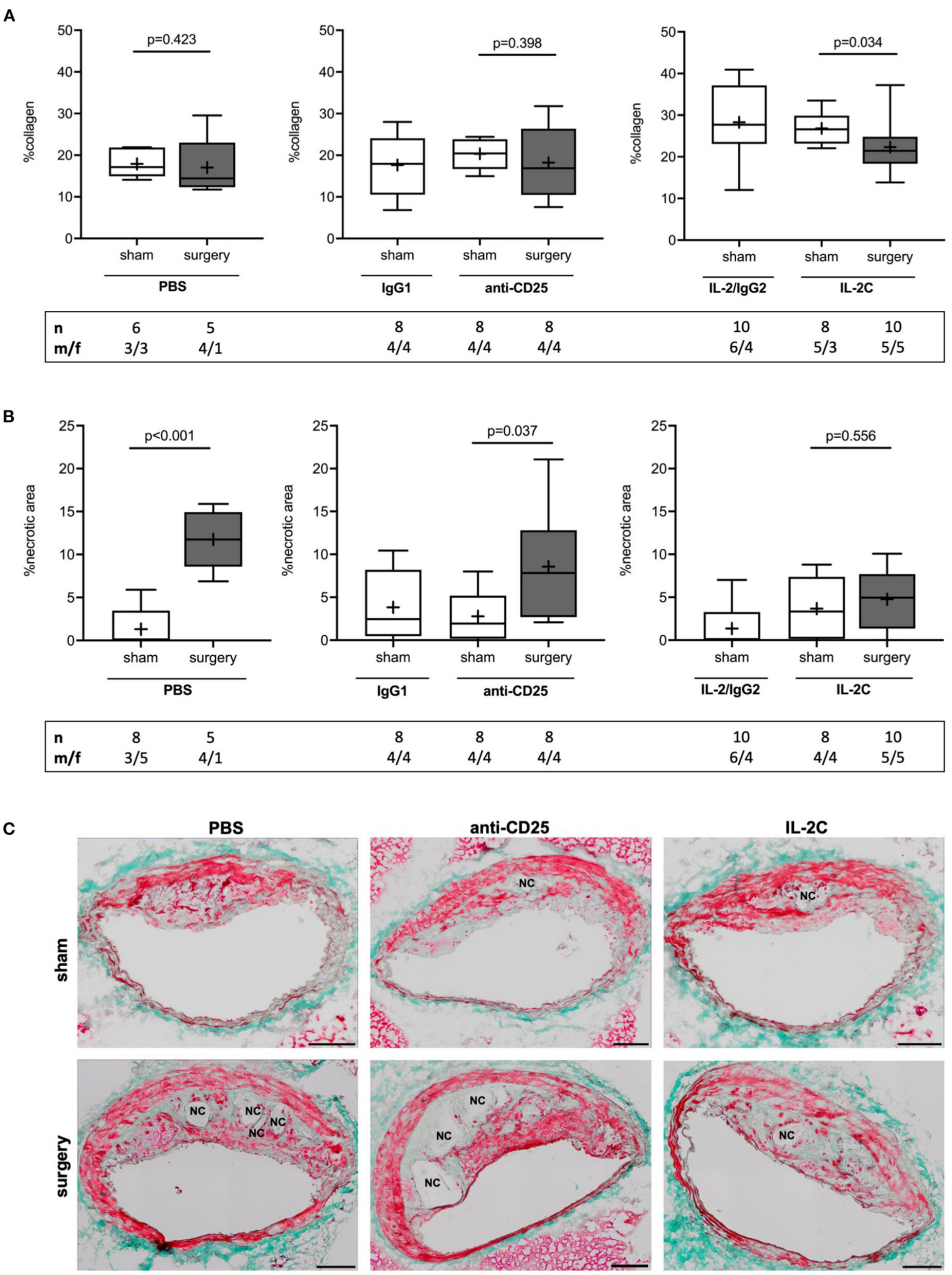
Effect of preoperatively modulated Treg levels on atherosclerotic plaque collagen content and perioperative necrotic core formation. Cross-sections of the brachiocephalic artery were stained with Masson trichrome to quantify **(A)** collagen content and **(B)** necrotic area relative to total plaque area at the site of maximum stenosis. **(C)** Representative images showing differences in perioperative necrotic core (NC) formation depending on the Treg-modulating treatment (scale bar 100 μm). The total number of mice under study as well as the ratio of male to female mice is indicated per group. Mice presenting with a maximum stenosis <10% and outliers identified by ROUT were excluded from the analysis resulting in *n* = 5–10 mice per group. Two-tailed Mann-Whitney U was used to compare sham and corresponding intervention groups; Kruskal-Wallis test was performed to assess differences between surgical intervention groups (gray; %collagen *p* = 0.258; % necrotic area *p* = 0.029).

Tregs are known to promote collagen synthesis (28), which largely contributes to plaque mechanical strength and resilience to shear stress (34). Collagen content was reduced by 20% in response to surgical stress in mice with preoperatively high Treg level only [IL-2C; 27 (23, 30) vs. 21 (18, 25) % for sham vs. surgery, *p* = 0.034]. However, no differences were observed among surgical intervention groups (Kruskal-Wallis *p* = 0.258). The comparison of sham groups showed the amount of collagen to be significantly increased by short-term IL-2C treatment when compared to PBS-injected animals [Kruskal-Wallis *p* = 0.001; 27 (23, 30) vs. 17 (15, 22) % collagen for IL-2C vs. PBS, *p* = 0.002] (Figure 4A). There were no sex-specific differences (Supplementary Figures 8A,B).

The presence and extent of NC area strongly correlates with atherosclerotic plaque vulnerability (35, 36). Assessment of lesional NC area showed an increase from 0.0 (0.0; 3.5) to 11 (5.2; 14) % in reponse to surgery in mice with normal preoperative Treg level (PBS; *p* < 0.001). Similarly, mice with preoperatively low Tregs showed a 7-fold increase of plaque necrosis after surgery [anti-CD25 mAb; 1.9 (0.2; 5.2) vs. 7.8 (2.7; 12.8) % for sham vs. surgery, *p* = 0.037]. This effect was absent in mice with preoperatively elevated Treg counts [IL-2C; 3.3 (0.1; 7.4) % vs. 5.0 (1.4; 7.7) %, *p* = 0.556]. Comparison of surgical treatment groups confirmed a statistical differenc among postoperative NC area of PBS and IL-2C-treated mice (Kruskal-Wallis *p* = 0.029; Dunn's *post-hoc* PBS vs. IL-2C: *p* = 0.025) (Figures 4B,C). Increase of NC area in PBS and anti-CD25 mAb-adminstered mice was present in both, male and female animals. Lack of perioperative NC formation in the IL-2C intervention group could mainly be attributed to female mice. However, compared to other treatment groups, NC area increase was rather small in male mice with preoperative high Treg count (Supplementary Figures 8C,D).

### Effect of Preoperative Treg Levels on Perioperative Lesional Macrophage Content

**Figure 5:**
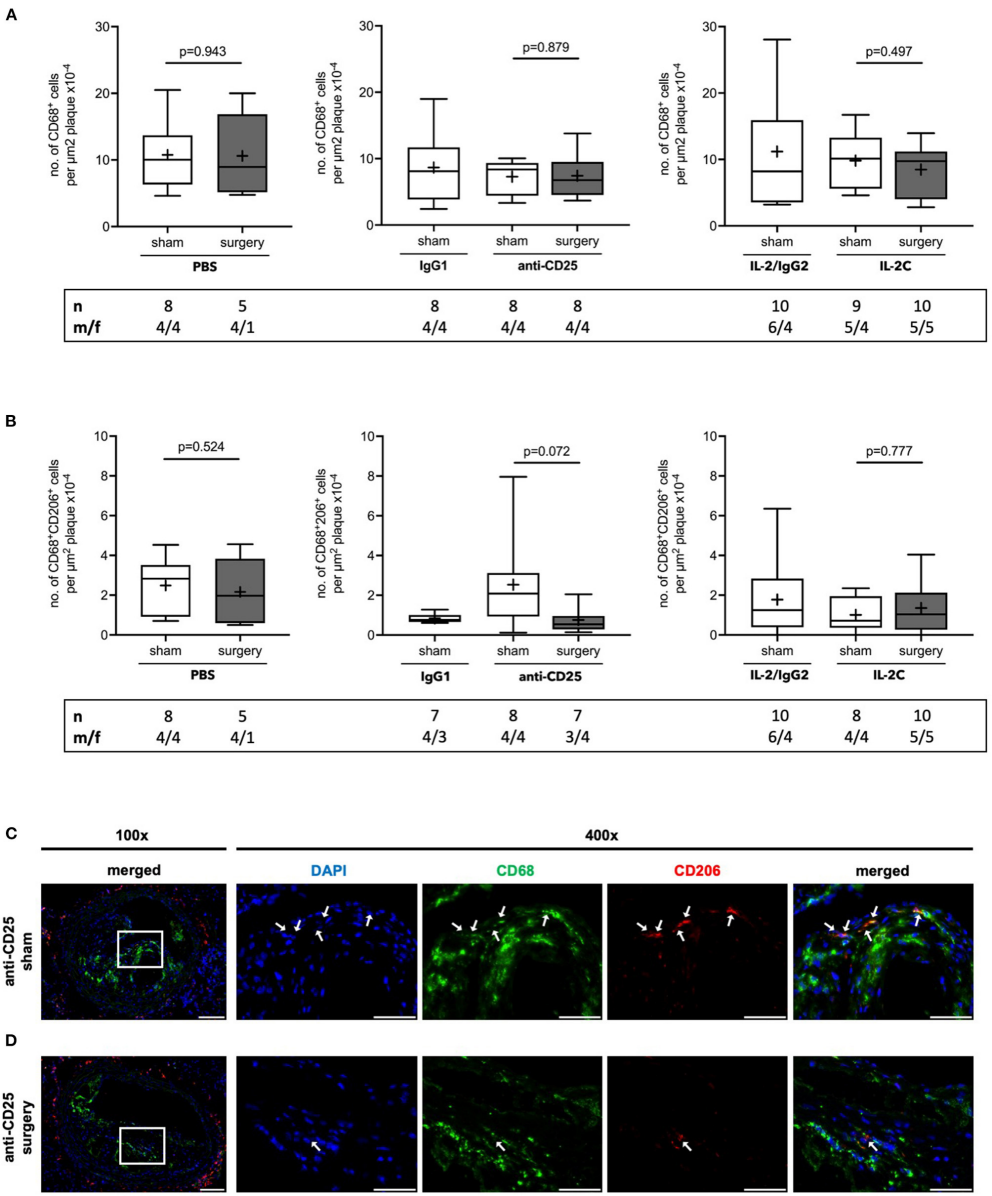
Quantification of atherosclerotic plaque macrophages in ApoE^−/−^ mice with modulated Treg levels and subjected to surgery or sham intervention. Immunofluorescent staining of **(A)** CD68^+^ total macrophages and **(B)** alternatively activated M2 subtype CD68^+^CD206^+^ macrophages per plaque area in cross-sections of brachiocephalic arteries at the site of maximum stenosis. **(C)** Representative micrographs of CD206 stainings in mice treated with anti-CD25 mAb and were subjected to sham or surgical intervention, respectively (scale bar 100 μm). High-magnification images are shown for box-indicated regions (scale bar 50 μm); arrows indicate CD68^+^CD206^+^ M2 macrophages. The total number of mice under study as well as the ratio of male to female mice is indicated per group. Mice presenting with a maximum stenosis <10% and outliers identified by ROUT were excluded from the analysis resulting in *n* = 5–10 mice per group. Two-tailed Mann-Whitney U was used to compare sham and corresponding intervention groups; Kruskal-Wallis test was used to assess differences between surgical intervention groups (gray; CD68^+^ total macrophages *p* = 0.625; CD68^+^CD206^+^ M2 macrophages *p* = 0.282).

As NC formation is closely related to macrophage apoptosis and efferocytosis, we next quantified the number of total and M2 subtype macrophages per plaque area. The number of lesional CD68^+^ total macrophages remained unchanged independent of preoperative Treg level and surgical stress (PBS: *p* = 0.943; anti-CD25 mAb: *p* = 0.879; IL-2C: *p* = 0.497) (Figure 5A). No statistical differences were observed in the number of alternatively activated, anti-inflammatory M2 macrophages (PBS: *p* = 0.524; IL-2C: *p* = 0.777). Though, mice with preoperatively low Tregs tended to develop decreased lesional M2 content in response to surgical stress [anti-CD25 mAb; 2.1 (0.9; 3.1) vs. 0.7 (0.3; 1.8) μm^2^ plaque area x10^−4^ for sham vs. surgery, *p* = 0.072] (Figures 5B,C). There was no difference between surgical intervention groups (Kruskal-Wallis *p* = 0.282). Observations were similar in both, male and female mice under study (Supplementary Figure 9).

### Preoperative Treg Modulation Does Not Affect Lesional SMC Content

**Figure 6:**
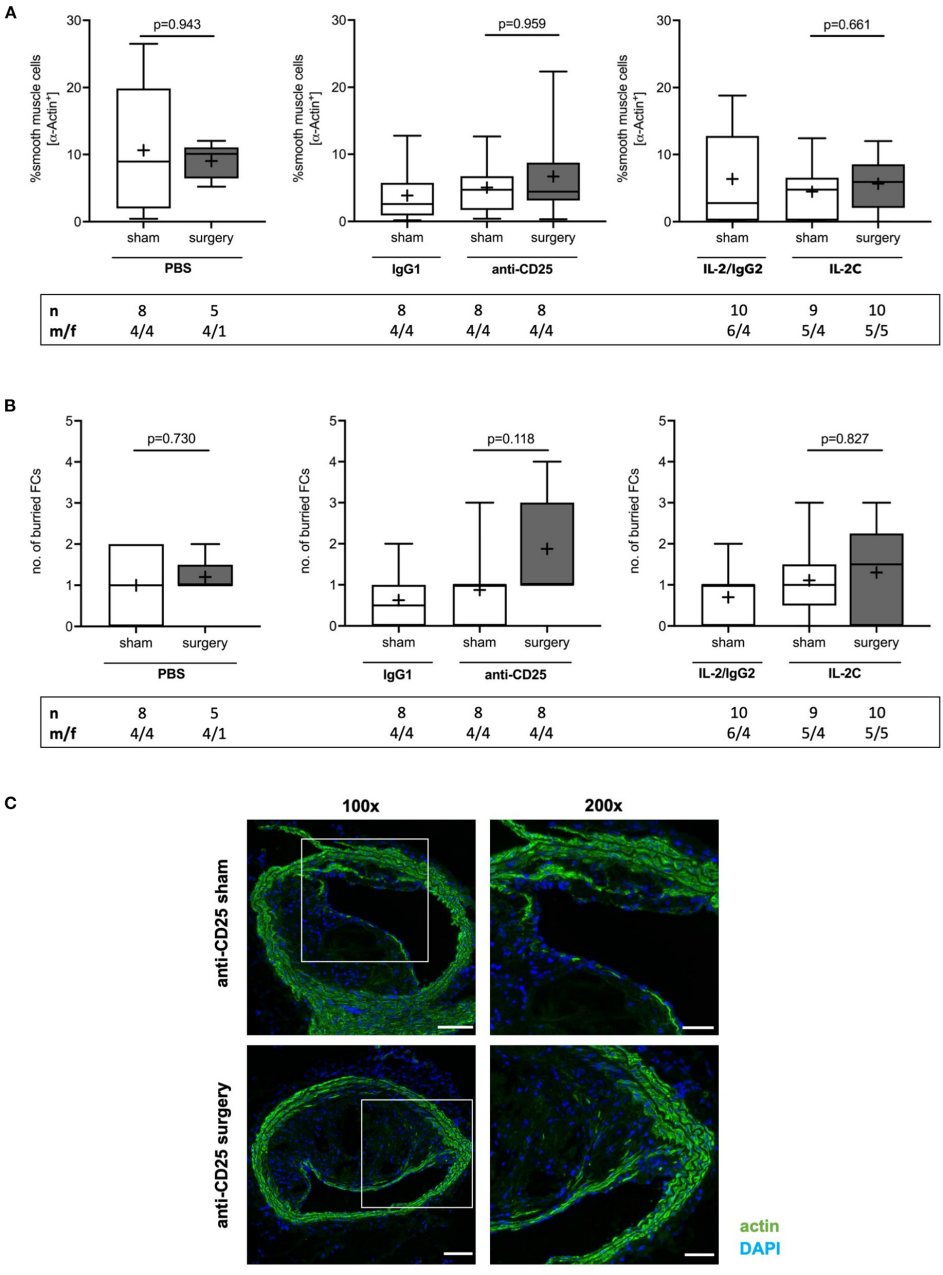
Effect of preoperative Treg levels on smooth muscle cell (SMC) content and number of buried fibrous caps (FC). **(A)** SMC content was quantified as the area staining positive for α smooth muscle actin relative to total plaque area. at the site of maximum stenosis **(B)** Lesional SMC-rich layers were identified as buried FC. **(C)** Immunofluorescent stainings showing representative images of buried FCs in mice treated with anti-CD25 mAb, that were subjected to sham or surgical intervention, respectively (scale bar 100 μm). High-magnification images are shown for box-indicated regions (scale bar 50 μm). The total number of mice under study as well as the ratio of male to female mice is indicated per group. Mice presenting with a maximum stenosis <10% and outliers identified by ROUT were excluded from the analysis resulting in *n* = 5–10 mice per group. Two-tailed Mann-Whitney U was used to compare sham and corresponding intervention groups; Kruskal-Wallis test was performed to assess differences between surgical intervention groups (gray; SMC content *p* = 0.178; buried FC *p* = 0.575).

SMCs represent the major source of extracellular matrix significantly promoting plaque stability in advanced atherosclerosis (37). Relative SMC content did not differ between groups (PBS: *p* = 0.943; anti-CD25 mAb: *p* = 0.959; IL-2C: *p* = 0.661) (Figure 6A) and independent of sex (Supplementary Figures 10A,B). Likewise, the number of buried FCs, possibly indicating previous episodes of plaque rupture (38), did not change in response to surgical stress and independent of the preoperative Treg level (PBS: *p* = 0.730; IL-2C: *p* = 0.827). Conversly, a trend toward an increase of buried FCs was seen in anti-CD25 mAb-treated mice with low Treg count with some plaques showing signs of up to four preceding rupture events (*p* = 0.118) (Figures 6B,C). Postoperative SMC content (Kruskal-Wallis *p* = 0.178) and the number of buried FCs (Kruskal-Wallis *p* = 0.575) did not differ between mice that underwent surgery. Overall, atherosclerotic plaques of female mice tended to display a lower number of buried FCs males (Supplementary Figures 10C,D).

### Increased Postoperative Plaque Stability in Mice With Preoperatively Upregulated Tregs

**Figure 7:**
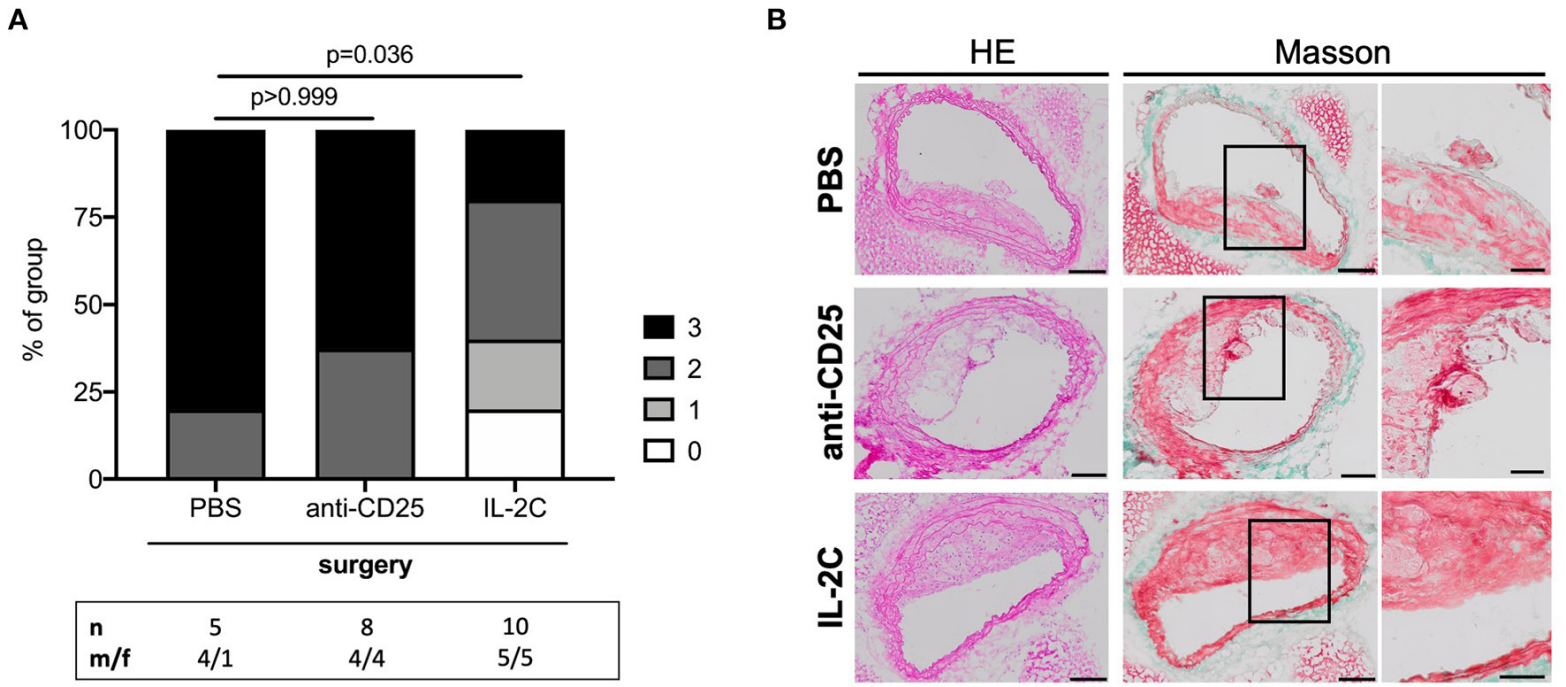
Post-surgical atherosclerotic plaque complexity stratified by preoperative Treg level. **(A)** Atherosclerotic plaques were scored and classified by complexity based on the presence of necrosis, buried fibrous caps and intraplaque hemorrhage at the site of maximum stenosis. If all criteria were met, plaques were assigned three points denoting the maximum complex lesion phenotype. The total number of mice under study as well as the ratio of male to female mice is indicated per group. Plaques with a maximum stenosis <10% were excluded from the analysis resulting in *n* = 5–10 mice per group. Kruskal-Wallis test (*p* = 0.024) followed by Dunn's *post-hoc* test were used to evaluate differences between PBS- and anti-CD25 mAb- or IL-2C-treated animals, respectively. **(B)** Representative micrographs of hematoxylin/eosin- and Masson trichrome-stained cross-sections derived from surgical intervention groups showing ruptured atherosclerotic plaques in mice treated with PBS and anti-CD25 mAb (scale bar 100 μm). High-magnification images are shown for box-indicated regions (scale bar 50 μm).

Atherosclerotic plaque complexity was further classified using a gradual score based on the presence or absence of necrosis, buried FCs, and intraplaque hemorrhage. There was no difference in postoperative lesion complexity between PBS- and anti-CD25 mAb-treated mice (*p* > 0.999). However, postoperative plaques of mice with high preoperative Treg levels presented with a more stable phenotype compared to controls (*p* = 0.036) (Figures 7A,B). This trend was present in both, male and female mice under study. However, male mice generally presented with a more complex plaque phenotype than females (Supplementary Figure 11).

## Discussion

Our data implicate regulatory T cell immunity in perioperative stress-driven rapid progression of atherosclerosis. In an atherosclerotic mouse model of surgical stress, we show that preoperative expansion of CD4^+^CD25^+^Foxp3^+^ Tregs by IL-2C treatment prevents from perioperative NC formation and enhances postoperative atherosclerotic plaque stability.

Impaired generation and immunosuppressive dysfunction of Tregs leads to dysregulated immune homeostasis and loss of tolerance (18), which critically drives atherosclerotic lesion initiation and progression (16). Correspondingly, patients with carotid atherosclerosis and acute coronary syndromes present with decreased numbers of Tregs (39–41), while low Treg frequencies positively correlate with long-term cardiovascular risk (24). In a prospective clinical study, we recently showed, that low preoperative Treg levels independently predict adverse cardiovascular outcome in coronary artery disease patients undergoing non-cardiac surgery (26).

The systemic stress response associated with surgical procedures is known to predispose to MI (42). Previous studies by our group revealed that mice exposed to perioperative stress display accelerated atherogenesis with increased postoperative lesion vulnerability. This effect is abrogated by pretreatment with an IL-6 blocking antibody, thereby inhibiting a perioperative inflammatory response (6). Our current data extent these findings by demonstrating that preoperatively expanded Tregs prevent from perioperative plaque destabilization mainly by limiting surgical stress-induced NC formation.

Ample evidence from experimental mouse studies indicates that Tregs actively modulate atherogenesis. Mice deprived of Tregs for a period of 4–8 weeks display exacerbated atherosclerotic plaque size and vulnerability (19, 20). Vice versa, adoptive transfer of Tregs reduces plaque burden, enhances lesion stability, and lowers the incidence of plaque disruption (21, 22, 28). Mechanistically, Tregs exert their atheroprotective function by inhibiting leukocyte recruitment (43) and foam cell formation (44), resolving inflammation (17), and by increasing SMC and collagen content (22) through cytokine-mediated and cell-cell contact-dependent mechanisms (45). Tregs further mediate numerous pro-resolving functions of monocyte-derived macrophages, which are ascribed a pivotal role in the pathogenesis of atherosclerosis.

Macrophages progressively accumulate within atherosclerotic lesions, release pro-inflammatory mediators, take up lipids and eventually give rise to foam cells, thereby exacerbating chronic inflammation of the arterial wall and significantly driving atherosclerotic plaque build-up (46). Defective clearance of apoptotic cells by macrophages, a process termed efferocytosis, results in NC formation and characterizes the transition from stable to unstable lesions (47). In murine plaques, a 2-fold increase of NC size is observed upon diphtheria toxin-induced depletion of Tregs (20). Mechanistic studies established a link between Tregs and efferocytotic capacity of macrophages in advanced atherosclerosis. Sharma et al. reported a Treg-dependent increase in efferocytosis and concomitant reduction of necrosis in regressing plaques (17). Moreover, *in vivo* expansion of Tregs for a period of 3 weeks enhances lesional macrophage efferocytosis in an IL-10-dependent manner without affecting lesion size and total macrophage content (48), which fits well with our observation reported here.

Notably, atherosclerotic lesions of mice treated with IL-2 displayed higher collagen content only 6 days after treatment initiation. Consistent with our finding, Tregs were reported to promote collagen deposition by increasing SMC proliferation and inhibiting extracellular matrix-degrading enzymes (22, 49). As expected for this short treatment period, we did not detect any changes in lesional SMC content. Therefore, we assume, that collagen enrichment likely resulted from reduced degradation by matrix metalloproteinases.

Human atherosclerotic plaques are histologically classified using the Stary Score. Based on the presence of qualitative characteristics, lesions are assigned to one of the six classes indicating the common sequence of lesion progression (I to VI). Plaques showing surface defects, thrombosis or hemorrhage are, however, collectively designated as type VI complicated lesions, which does not provide a sufficiently high resolution to assess differences in plaque vulnerability (31). Calculation of a morphology score by separately grading the presence or absence of necrotic area, buried FCs and intraplaque hemorrhage, allowed us to create a holistic picture of postoperative plaque vulnerability (6). Here, mice with preoperatively high Treg levels presented with more stable plaques compared to PBS-treated control mice, suggesting a protective effect of preoperative Treg expansion. Other markers of plaque instability, like lesional macrophage content and the number of buried FCs were not affected by preoperatively modulated Treg levels. Accelerated atherosclerosis has also been observed in a murine model of orthopedic surgery. Atherosclerotic plaques of the aortic root were found to be significantly increased mainly due to enlargement of necrotic area at 15 days post-surgery, but not before (7). Given that the majority of cardiovascular events following major non-cardiac surgery occurs within the first couple of days after surgery (8), our mice were sacrificed 3 days after the surgical insult. Though, it remains possible, that the effect of preoperatively modulated Treg levels on perioperative atherogenesis would become more pronounced at later time points.

Observed effects appeared to be largely independent of sex. Although the study was not sufficiently powered to allow direct statistical comparison of sex as an independent variable, sex-stratified data shown in the Supplementary Material of this article suggest male mice to display a generally higher plaque burden and instability compared to female littermates. This generally fits the situation in patients, where plaque size tends to be greater in males, particularly at younger ages. It was suggested, that sex hormones and sex chromosomes may actively modulate immune responses and thus, susceptibility to inflammatory diseases (50). Here, we found diminished perioperative plaque destabilization in mice with preoperatively elevated Treg levels to be present in both sexes.

Treatment with anti-CD25 mAb reduced, but did not eliminate CD4^+^CD25^+^Foxp3^+^ cells. In addition, antibody-dependent modulation of Tregs was less effective compared to C57BL/6 wild type animals (51, 52), which may be attributed to compromised Treg number and function in atherosclerotic ApoE^−/−^ mice (53). Future studies will show, whether adoptive transfer or total depletion of Tregs using an inducible transgenic mouse model will have a more significant effect on perioperative plaque destabilization.

Our study comes with some important limitations. Perioperative stress was inflicted by a combination of laparotomy and hemorrhage, which renders the model well generalizable, but unspecific. Moreover, we did not replace bloodshed as it would be in the clinical setting. Yet, heart rate and blood pressure monitoring within previous studies of our group revealed, that the intervention induces mild hemodynamic strain similar to the clinical scenario of major surgery, rather than hemorrhagic shock (6). Depletion of Tregs was induced by treatment with anti-CD25 mAb, which specifically recognizes the IL-2 receptor α subunit. IL-2 signaling is essential for the generation, peripheral proliferation and maintenance of Tregs (54). However, activated effector T cells transiently express CD25, likewise (55). Although we did not observe any significant changes in any leukocyte population other than Tregs, we cannot fully exclude that other CD25-expressing cells were affected by our treatment and thus contributed to the observed phenotype. During decades, murine models have served as valuable tools to study experimental atherosclerosis due to their rapid reproduction, ease of genetic manipulation and the ability to control for environmental risk factors (56). Although sharing many features, murine plaques differ in the incidence of thrombotic occlusion and mechanical stress, limiting direct extrapolation to human atherosclerosis (57).

Together, the here presented data in connection with our clinical observation (26) support that therapeutic expansion or restoration of Tregs prior to major surgery may hold promise to dampen perioperative stress-induced atherosclerotic plaque destabilization thereby lowering the risk of perioperative cardiovascular events. Of note, low-dose IL-2-mediated expansion of Tregs is currently being investigated in a phase I/II clinical trial for prevention of recurrent MI in patients with stable ischemic disease or acute coronary syndromes (58). Only recently, the international, randomized CANTOS trial convincingly demonstrated the benefit of anti-inflammatory therapy on the risk of recurrent cardiovascular events in post-MI patients with residual inflammatory risk (59). Our study in mice will stimulate further research into strategies to restrain perioperative inflammation by enhancing Treg function and number in order to reduce cardiovascular risk.

## Data Availability Statement

The raw data supporting the conclusions of this article will be made available by the authors, without undue reservation.

## Ethics Statement

The animal study was reviewed and approved by Regierungspräsidium Karlsruhe.

## Author Contributions

JH and LK collected samples and performed experiments. JH analyzed data. JH, MW, and JL interpreted the data. JL designed the project. JH and JL drafted the manuscript. LK and MW revised the manuscript. All authors read and approved the final manuscript.

## Conflict of Interest

The authors declare that the research was conducted in the absence of any commercial or financial relationships that could be construed as a potential conflict of interest.

## Publisher's Note

All claims expressed in this article are solely those of the authors and do not necessarily represent those of their affiliated organizations, or those of the publisher, the editors and the reviewers. Any product that may be evaluated in this article, or claim that may be made by its manufacturer, is not guaranteed or endorsed by the publisher.
