# Supplementary material for: Modulation of Peripheral CD4+CD25+Foxp3+ Regulatory T Cells Ameliorates Surgical Stress-Induced Atherosclerotic Plaque Progression in ApoE-Deficient Mice
Source: Front Cardiovasc Med. 2021 Aug 12;8:682458. doi: 10.3389/fcvm.2021.682458 (PMC8416168; doi:10.3389/fcvm.2021.682458)
Supplement: Supplementary file 1 [file Data_Sheet_1.pdf]

Supplementary Material

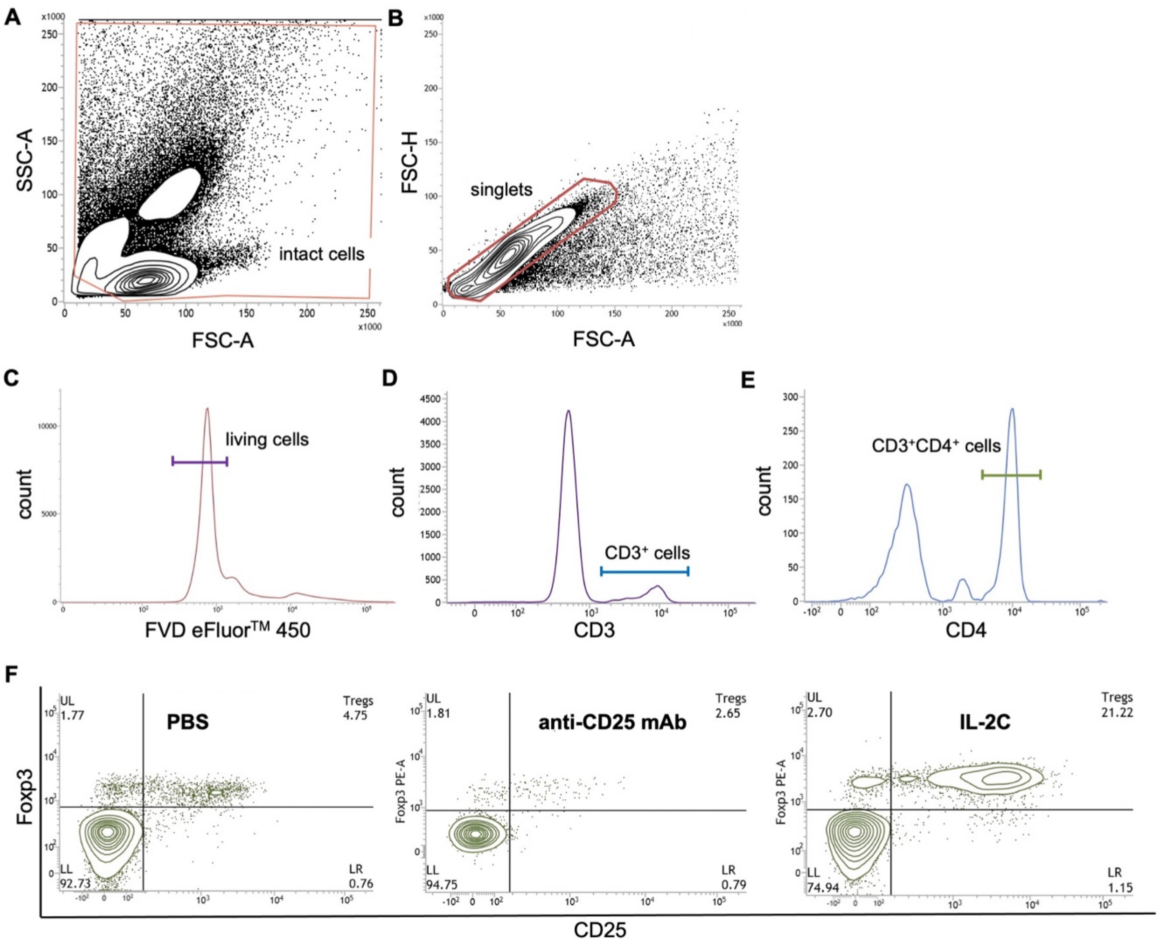

Supplementary Figure S1. Gating strategy for the flow cytometric quantification of regulatory T cells from murine blood and lymphoid organs. (A) After exclusion of cell debris and (B) selection of singlets, (C) living cells were selected for simultaneous surface expression of (D) CD3 and (E) CD4. (F) Identification of Tregs was based on their expression of CD25 and Foxp3. Representative Treg counts of mice either treated with PBS, CD25 mAb or IL-2C are given.

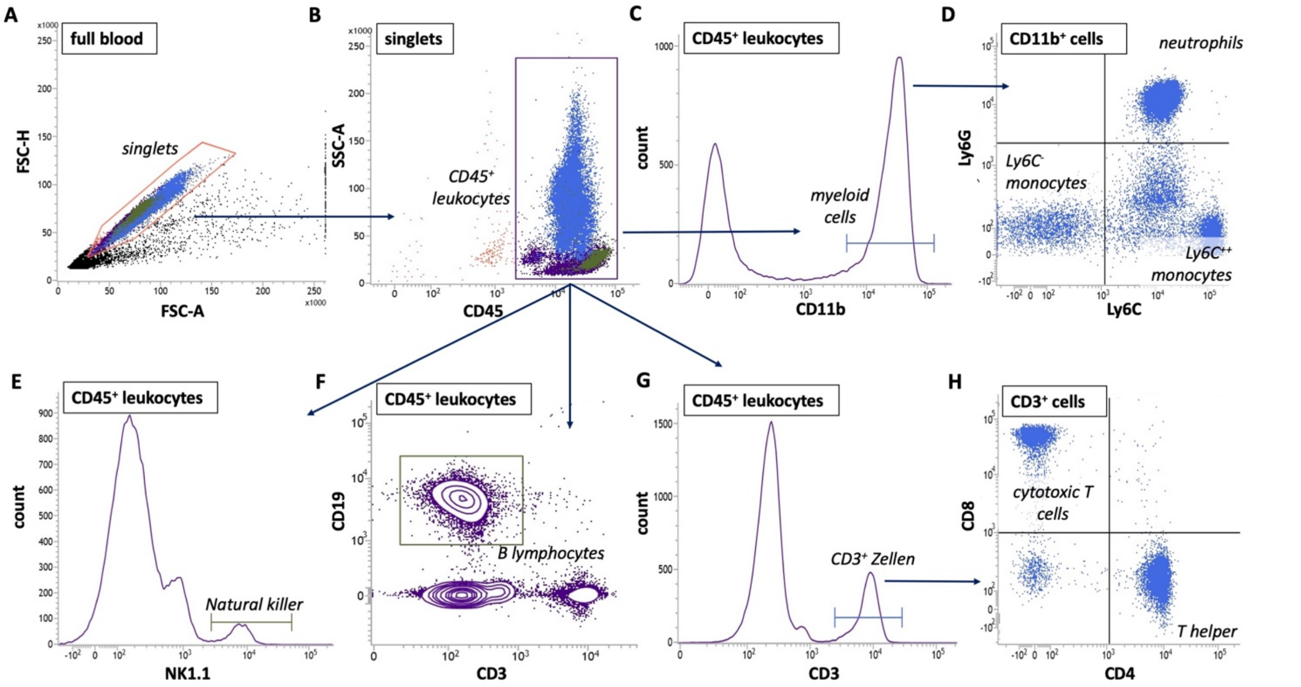

Supplementary Figure S2. Gating strategy for the flow cytometric quantification of leukocyte subpopulation from full blood samples. (A) After exclusion of cell clumps, (B) leukocytes were selected based on their CD45 expression. (C) CD11-expressing myeloid cells (D) were analyzed for the expression of Ly6C and Ly6G. (E) CD45-expressing leukocytes were further evaluated for their expression of NK1.1. (F) B cells were defined as $CD3^{-}CD19^{+}$ expressing cells. (G) Cells staining positive for CD3 (H) were analyzed for the presence of CD4 and CD8 to discriminate between T helper and cytotoxic T cells.

A

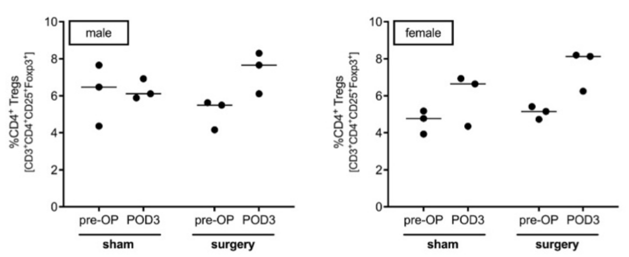

		male sham pre-OP	male sham POD3	male surgery pre-OP	male surgery POD3	female sham pre-OP	female sham POD3	female surgery pre-OP	female surgery POD3
**median**		6.5	6.1	5.5	7.7	4.8	6.6	5.2	8.1
**IQR**	**25th**	4.4	5.9	4.2	6.1	3.0	4.4	4.7	6.3
	**75th**	7.7	6.9	5.6	8.3	5.2	6.9	5.4	8.2
**mean**		6.2	6.3	5.1	7.4	4.6	6.0	5.1	7.5
**SD**		1.7	0.5	0.8	1.1	0.6	1.4	0.3	1.1

B

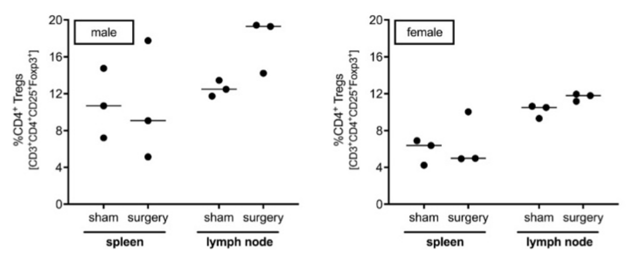

		male spleen sham	male spleen surgery	male lymph node sham	male lymph node surgery	female spleen sham	female spleen surgery	female lymph node sham	female lymph node surgery
**median**		11	9.1	12	19	11	5.0	11	12
**IQR**	**25th**	7.2	5.1	12	14	9.3	4.9	9.3	11
	**75th**	15	18	13	19	11	10	11	12
**mean**		11	11	13	18	10	6.7	10	12
**SD**		3.8	6.5	0.9	3.0	0.7	2.9	0.7	0.4

C

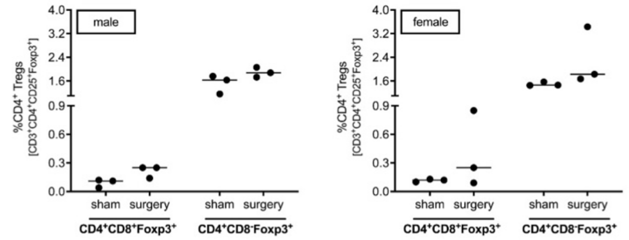

		male CD4+CD8+Foxp3+ sham	male CD4+CD8+Foxp3+ surgery	male CD4+CD8-Foxp3+ sham	male CD4+CD8-Foxp3+ surgery	female CD4+CD8+Foxp3+ sham	female CD4+CD8+Foxp3+ surgery	female CD4+CD8-Foxp3+ sham	female CD4+CD8-Foxp3+ surgery
**median**		0.11	0.25	1.63	1.88	0.12	0.25	1.46	1.83
**IQR**	**25th**	0.04	0.14	1.16	1.73	0.10	0.09	1.45	1.67
	**75th**	0.12	0.25	1.76	2.06	0.13	0.85	1.57	3.43
**mean**		0.09	0.21	1.52	1.89	0.17	0.40	1.49	2.3
**SD**		0.04	0.06	0.32	0.17	0.02	0.40	0.07	0.97

D

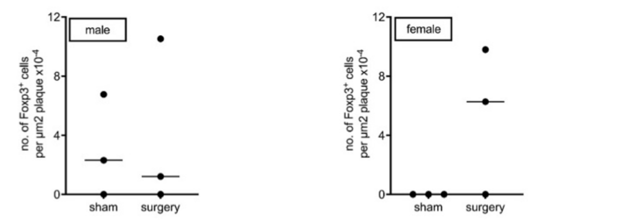

		male sham	male surgery	female sham	female surgery
**median**		2.3	1.2	0.0	6.3
**IQR**	**25th**	0.0	0.0	0.0	0.0
	**75th**	6.8	11	0.0	9.8
**mean**		3.0	3.9	0.0	5.4
**SD**		3.4	5.8	0.0	5.0

Supplementary Figure S3. Sex-stratified data showing the effect of surgery on regulatory T cell counts in (A) blood, (B) spleen, lymph node, (C) thymus, (D) and atherosclerotic plaques.

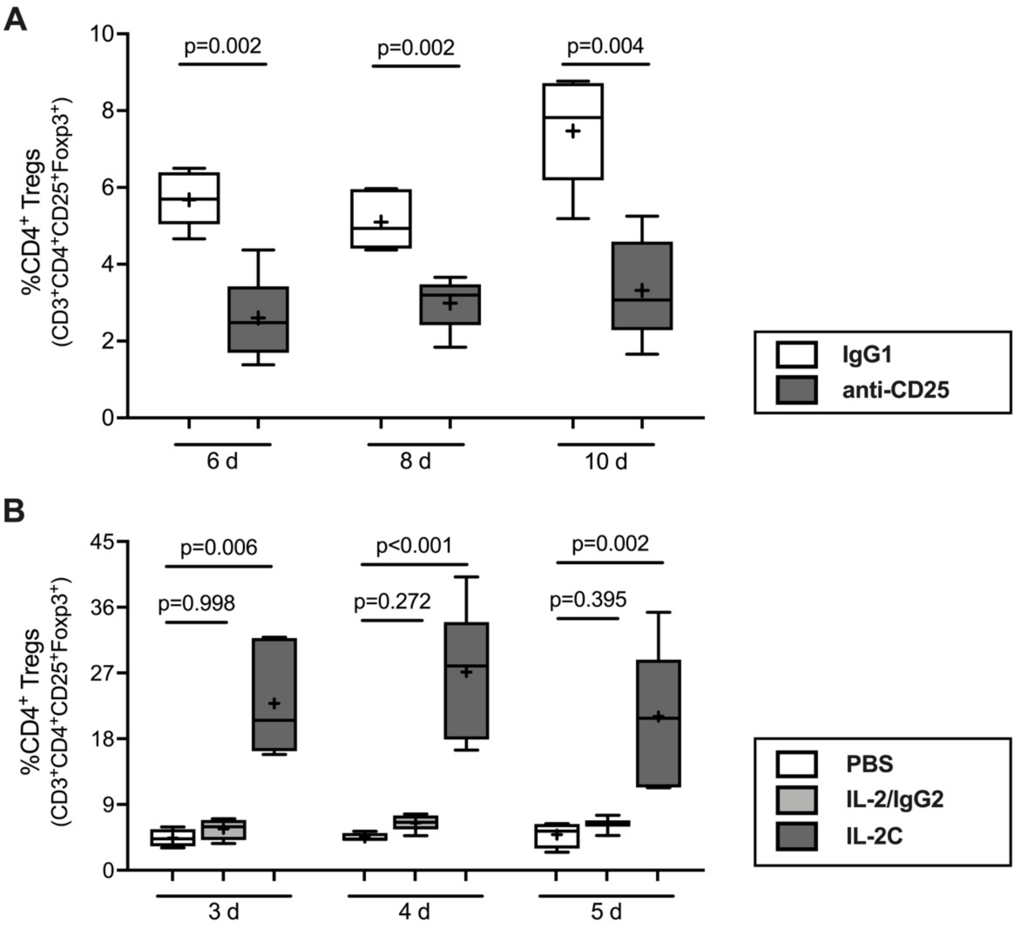

Supplementary Figure S4. Establishment of peripheral CD4$^+$CD25$^+$Foxp3$^+$ Treg modulation in atherosclerotic ApoE$^{-/-}$ mice. CD4$^+$CD25$^+$Foxp3$^+$ Treg were quantified by flow cytometry at three different time points after treatment initiation. (A) For Treg reduction, atherosclerotic ApoE$^{-/-}$ mice (n=6 per group; 3m, 3f) were injected with monoclonal anti-CD25 or isotype matched control antibody IgG1. Differences between groups were assessed using non-parametric Mann-Whitney U test. (B) Expansion of Tregs was induced using a complex of IL-2/anti-IL-2 (n=6; 3m, 3f). An isotype matching control antibody (IgG2; n=6; 3m, 3f) and PBS (n=4; 2m, 2f) were used as controls. Differences between the three treatment groups were assessed using non-parametric Kruskal-Wallis test followed by Dunn's multiple comparison test to compare blood Treg counts from IL-2/IgG2- and IL-2C-treated mice to those of the PBS group per time point.

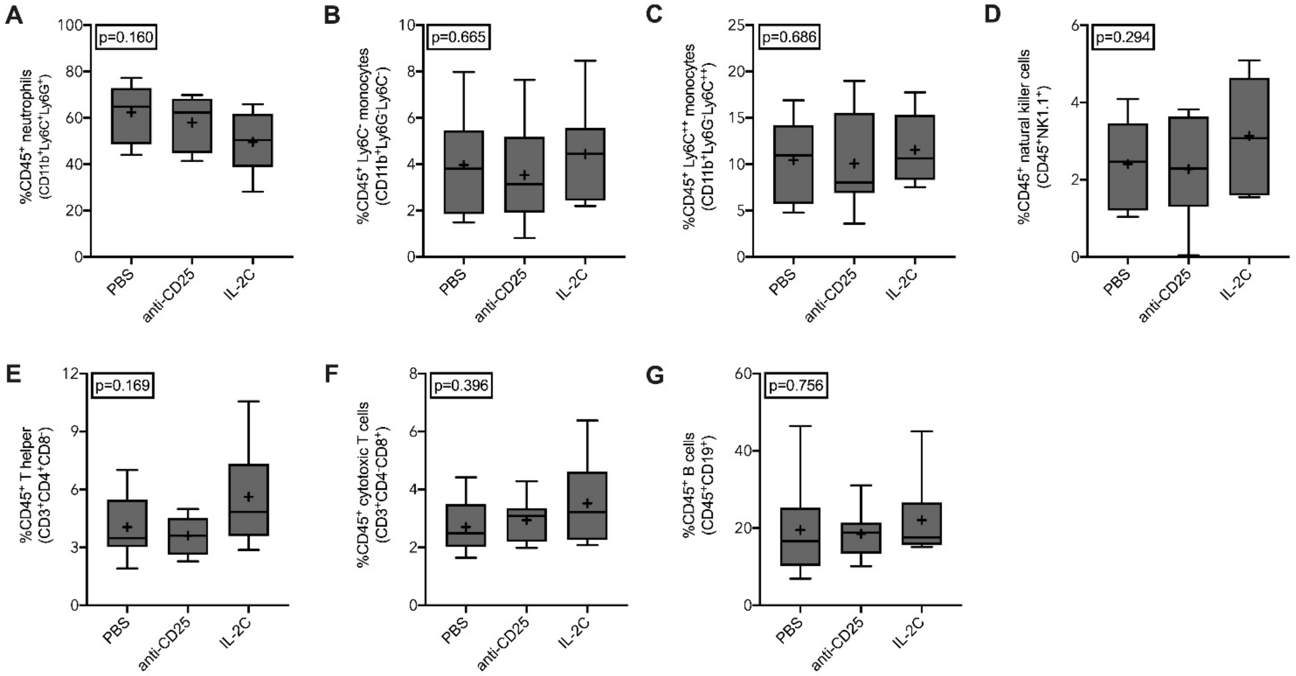

Supplementary Figure S5. Flow cytometric quantification of leukocyte subpopulations in blood of atherosclerotic ApoE$^{-/-}$ mice treated with PBS (n=8; 4m, 4f), monoclonal anti-CD25 antibody (n=8; 4m, 4f) and IL-2/anti-IL-2 complex (IL-2C; n=8; 4m, 4f). (A-C) Myeloid and (D-G) lymphoid cell populations were quantified in blood samples obtained right before skin incision to evaluate the specificity of the Treg modulating treatment. Data were expressed relative to CD45^{+} total leukocytes. Differences were assessed using Kruskal-Wallis test. Applying Bonferroni correction, $p < 0.007$ (0.05/7) was considered statistically significant.

A

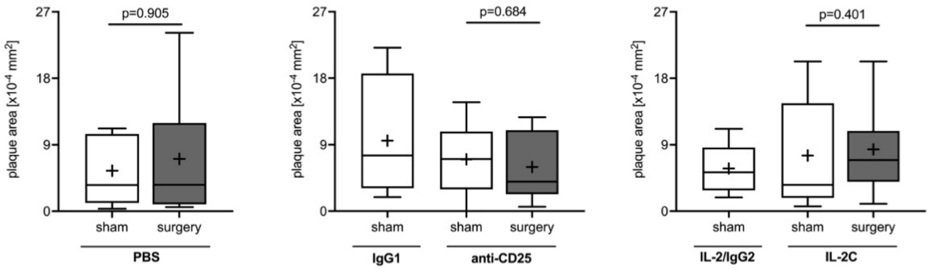

**n**	10	9	8	10	10	10	11	11
**m/f**	4/6	4/5	4/4	4/6	4/6	6/4	5/6	5/6

B

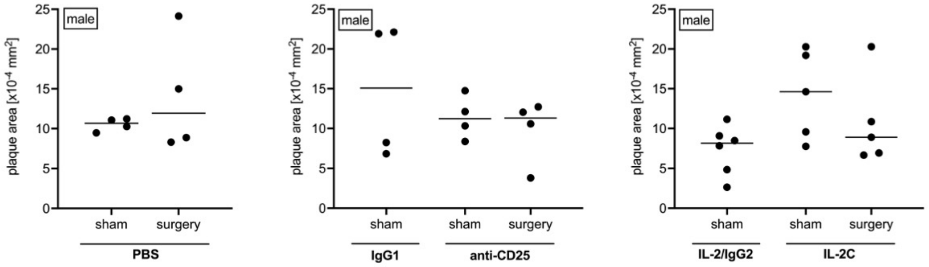

**median**		11	12	15	11	11	8.2	14	8.9
**IQR**	**25th**	9.7	8.4	7.2	8.9	5.5	4.3	8.7	6.8
	**75th**	11	22	22	14	13	9.6	19	16
**mean**		11	14	15	11	9.8	7.3	14	11
**SD**		0.8	7.4	8.4	2.7	4.1	3.1	5.6	5.6

C

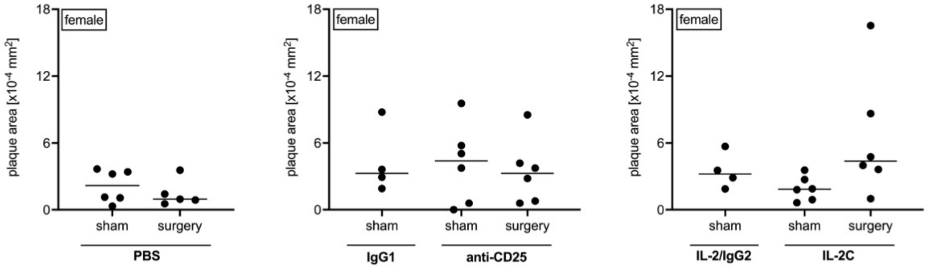

**median**		2.2	1.0	3.3	4.4	3.3	3.2	1.9	4.4
**IQR**	**25th**	0.9	0.7	2.2	0.4	0.7	2.1	0.8	3.0
	**75th**	3.5	2.5	7.5	6.7	5.3	5.2	2.9	11
**mean**		2.1	1.5	4.1	4.1	3.4	3.5	1.9	6.4
**SD**		1.5	1.2	3.5	3.5	2.9	1.6	1.1	5.5

Supplementary Figure S6. Effect of preoperative regulatory T cell levels on perioperative plaque area at the site of maximum stenosis for (A) all mice under study, (B) male, and (C) female mice. For (A), outliers were excluded using the ROUT method; group comparisons were made using non-parametric Mann-Whitney U test.

A

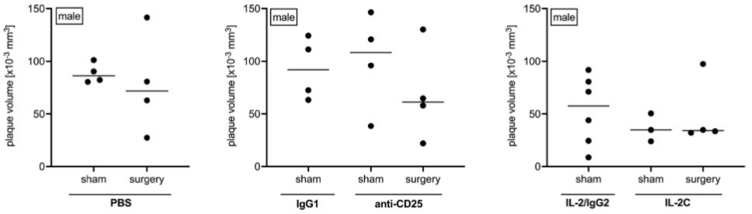

**median**		86.2	71.8	91.9	108	61.3	57.5	34.7	34.0
**IQR**	**25th**	80.9	36.2	65.6	52.8	30.9	20.4	23.9	32.3
	**75th**	98.5	126	121	140	114	83.3	50.4	81.4
**mean**		88.5	78.1	92.8	100	68.7	53.4	36.3	49.4
**SD**		9.48	47.8	29.6	46.2	45.1	33.0	13.4	32.1

B

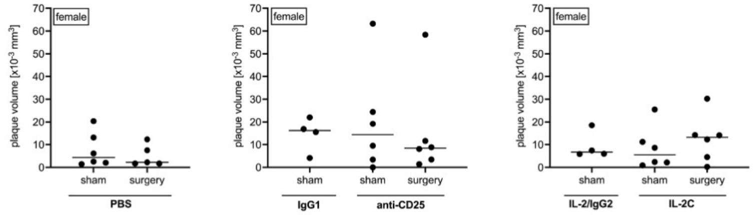

**median**		4.35	2.25	16.2	14.35	8.47	6.71	5.49	13.2
**IQR**	**25th**	1.91	1.72	6.98	2.56	2.96	5.91	1.87	3.48
	**75th**	15.0	9.93	20.7	34.14	23.3	15.8	14.8	18.2
**mean**		7.63	5.11	14.6	20.0	15.3	9.47	8.47	12.6
**SD**		7.62	4.72	7.54	23.1	21.4	6.10	9.29	10.3

C

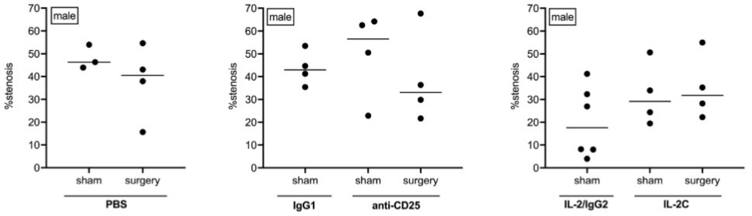

**median**		46	41	43	57	33	18	29	32
**IQR**	**25th**	44	21	37	30	24	7.0	21	24
	**75th**	54	52	51	64	60	35	46	50
**mean**		48	38	44	50	39	20	32	35
**SD**		5.3	16	7.5	19	20	15	14	14

D

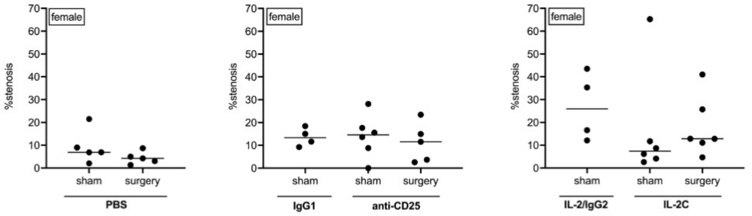

**median**		6.9	4.2	13	15	12	26	7.4	13
**IQR**	**25th**	4.5	2.2	9.9	6.6	3.1	13	3.8	9.5
	**75th**	15	6.8	18	20	19	41	25	30
**mean**		9.3	4.4	14	14	11	27	16	18
**SD**		7.3	2.7	4.0	9.4	8.6	15	24	13

Supplementary Figure S7. Effect of preoperative regulatory T cell levels on perioperative (A, B) atherosclerotic plaque volume and (C, D) relative stenosis stratified by sex.

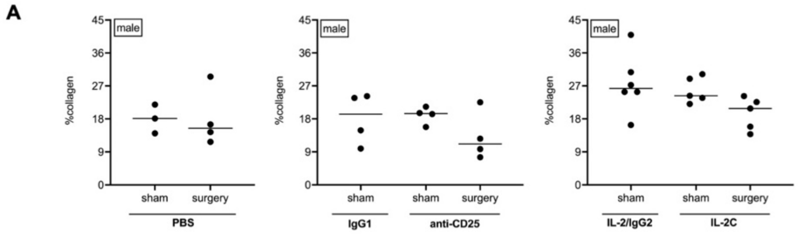

		PBS sham	PBS surgery	IgG1 sham	anti-CD25 sham	anti-CD25 surgery	IL-2/IgG2 sham	IL-2C sham	IL-2C surgery
median		18	15	19	19	11	26	24	21
IQR	25th	14	12	11	17	8.1	23	23	15
	75th	22	26	24	21	20	33	30	23
mean		18	18	18	19	13	28	26	19
SD		3.9	7.9	7.0	2.3	6.6	8.1	3.5	4.4

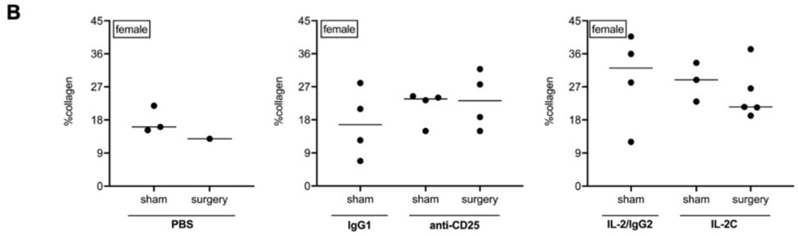

		PBS sham	PBS surgery	IgG1 sham	anti-CD25 sham	anti-CD25 surgery	IL-2/IgG2 sham	IL-2C sham	IL-2C surgery
median		16	13	17	24	23	32	29	22
IQR	25th	15	13	8.2	17	16	16	23	20
	75th	22	13	26	24	31	40	34	32
mean		18	13	17	22	23	29	28	25
SD		3.6	0.0	9.3	4.5	7.8	13	5.3	7.3

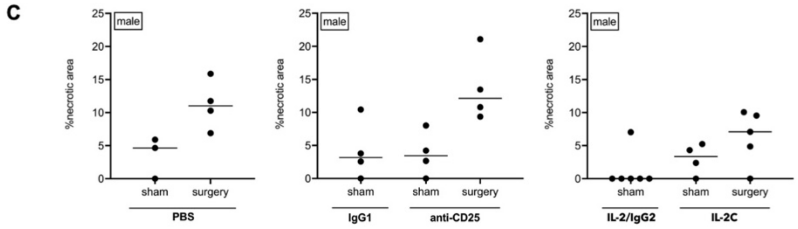

		PBS sham	PBS surgery	IgG1 sham	anti-CD25 sham	anti-CD25 surgery	IL-2/IgG2 sham	IL-2C sham	IL-2C surgery
median		4.6	11	3.2	3.4	12	0.0	3.3	7.1
IQR	25th	0.0	7.7	0.6	0.7	9.7	0.0	0.6	2.4
	75th	5.9	15	8.8	7.1	19	1.8	5.0	9.8
mean		3.5	11	4.2	3.7	14	1.2	3.0	6.3
SD		3.1	3.7	4.4	3.3	5.2	2.9	2.3	4.1

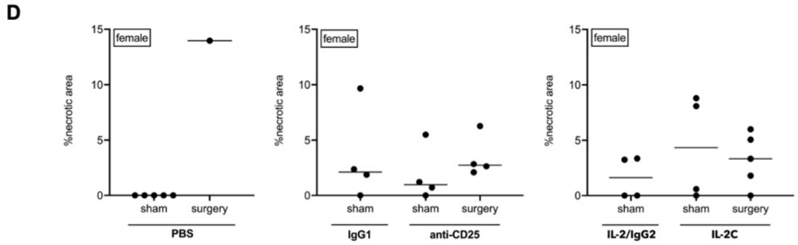

		PBS sham	PBS surgery	IgG1 sham	anti-CD25 sham	anti-CD25 surgery	IL-2/IgG2 sham	IL-2C sham	IL-2C surgery
median		0.0	14	2.1	1.0	2.7	1.6	4.3	3.3
IQR	25th	0.0	14	0.5	0.2	2.2	0.0	0.1	0.9
	75th	0.0	14	7.8	4.4	5.4	3.3	8.6	5.5
mean		0.0	14	3.5	1.9	3.5	1.7	4.4	3.2
SD		0.0	0.0	4.3	2.5	1.9	1.9	4.7	2.4

Supplementary Figure S8. Effect of preoperative regulatory T cell levels on perioperative (A, B) atherosclerotic plaque collagen content and (C, D) relative necrotic core area stratified by sex.

A

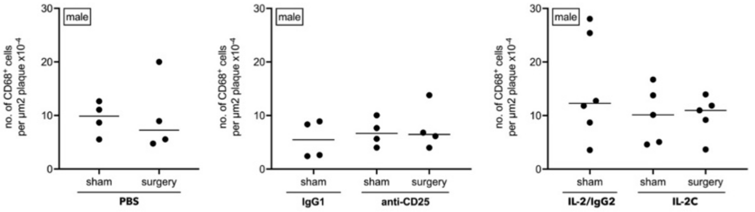

**median**		9.9	7.3	5.5	6.7	6.5	12	10	11
**IQR**	**25th**	6.3	5.0	2.5	4.4	4.5	7.4	4.8	6.4
	**75th**	12	17	8.8	9.5	12	26	15	13
**mean**		9.5	9.8	5.6	6.8	7.7	15	10	9.9
**SD**		3.1	7.0	3.5	2.6	4.2	9.6	5.3	3.9

B

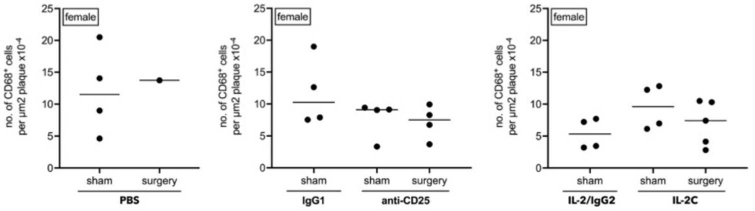

**median**		12	14	10	9.1	7.5	5.3	9.6	7.4
**IQR**	**25th**	5.7	14	7.6	4.8	4.4	3.3	6.3	3.5
	**75th**	19	14	17	9.3	9.5	7.6	13	10
**mean**		12	14	12	7.7	7.1	5.4	9.5	7.0
**SD**		6.8	0	5.4	2.9	2.7	2.4	3.5	3.5

C

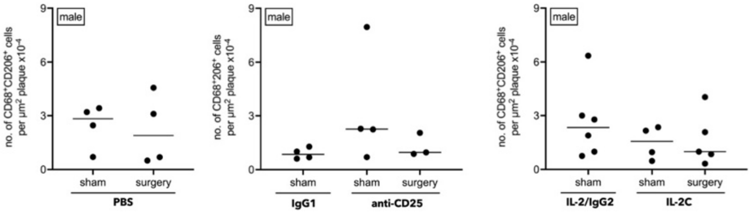

**median**		2.8	1.9	0.9	2.3	1.0	2.3	1.6	1.0
**IQR**	**25th**	1.1	0.5	0.6	1.1	0.9	0.9	0.6	0.6
	**75th**	3.4	4.2	1.2	6.5	2.1	3.8	2.3	3.1
**mean**		2.4	2.2	0.9	3.3	1.3	2.6	1.5	1.7
**SD**		1.2	2.0	0.3	3.2	0.7	2.0	0.9	1.5

D

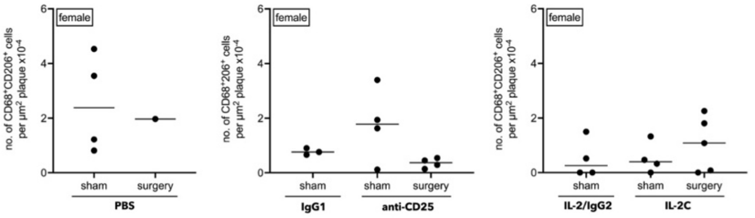

**median**		2.4	2.0	0.8	1.8	0.4	0.3	0.4	1.1
**IQR**	**25th**	0.9	2.0	0.7	0.5	0.2	0.0	0.1	0.0
	**75th**	4.3	2.0	0.9	3.0	0.5	1.3	1.1	2.0
**mean**		2.5	2.0	0.8	1.8	0.4	0.5	0.5	1.0
**SD**		1.8	0.0	0.1	1.3	0.2	0.7	0.6	1.0

Supplementary Figure S9. Effect of preoperative regulatory T cell levels on (A, B) total macrophage and (C, D) alternatively activated M2 macrophage content of atherosclerotic plaques stratified by sex.

A

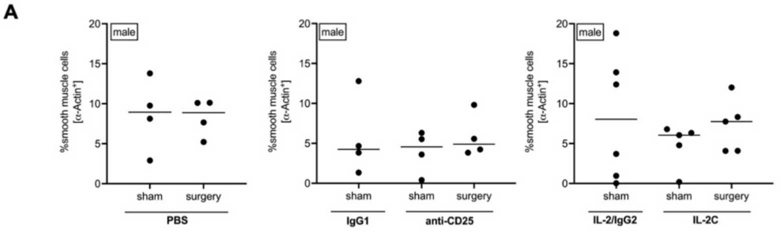

**median**		8.9	8.8	4.2	4.6	4.9	8.0	6.0	7.8
**IQR**	**25th**	4.2	5.8	2.0	1.2	3.9	0.7	2.5	4.1
	**75th**	13	10	11	6.1	8.7	15	6.6	10
**mean**		8.6	8.3	5.7	4.0	5.9	8.3	4.8	7.2
**SD**		4.5	2.3	5.0	2.6	2.7	7.8	2.7	3.3

B

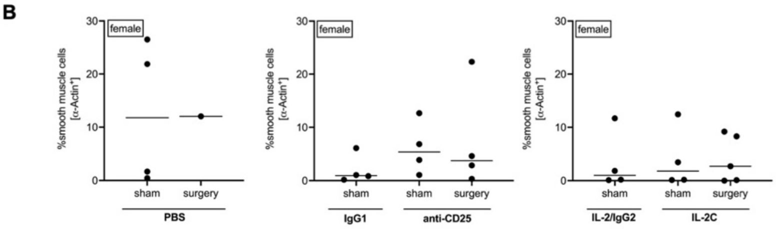

**median**		12	12	1.0	5.4	3.7	1.0	1.8	2.7
**IQR**	**25th**	0.7	12	0.3	1.8	1.0	0.1	0.1	0.1
	**75th**	25	12	4.8	11	18	9.2	10	8.8
**mean**		13	12	2.0	6.1	7.5	3.5	4.0	4.1
**SD**		14	0.0	2.7	5.0	10	5.6	5.8	4.4

C

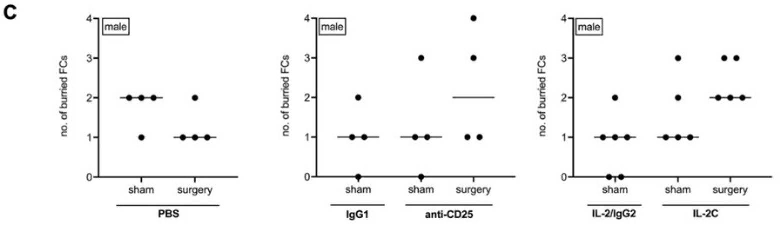

**median**		2.0	1.0	1.0	1.0	2.0	1.0	1.0	2.0
**IQR**	**25th**	1.3	1.0	0.3	0.3	1.0	0.0	1.0	2.0
	**75th**	2.0	1.8	1.8	2.5	3.8	1.3	2.5	3.0
**mean**		1.8	1.3	1.0	1.3	2.3	0.8	1.6	2.4
**SD**		0.5	0.5	0.8	1.3	1.5	0.8	0.9	0.5

D

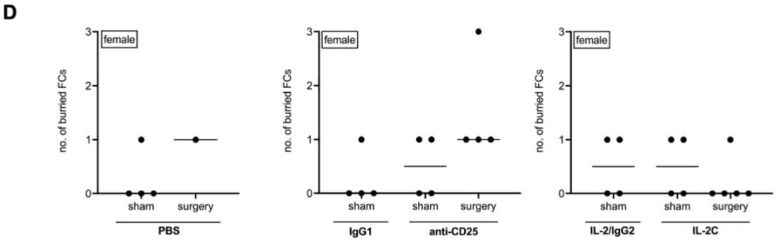

**median**		0.0	1.0	0.0	0.5	1.0	0.5	0.5	0.0
**IQR**	**25th**	0.0	1.0	0.0	0.0	1.0	0.0	0.0	0.0
	**75th**	0.8	1.0	0.8	1.0	2.5	1.0	1.0	0.5
**mean**		0.3	1.0	0.3	0.5	1.5	0.5	0.5	0.2
**SD**		0.5	0.0	0.5	0.6	1.0	0.6	0.6	0.4

Supplementary Figure S10. Effect of preoperative regulatory T cell levels on perioperative (A, B) atherosclerotic plaque smooth muscle cell content and (C, D) the number of buried fibrous caps stratified by sex.

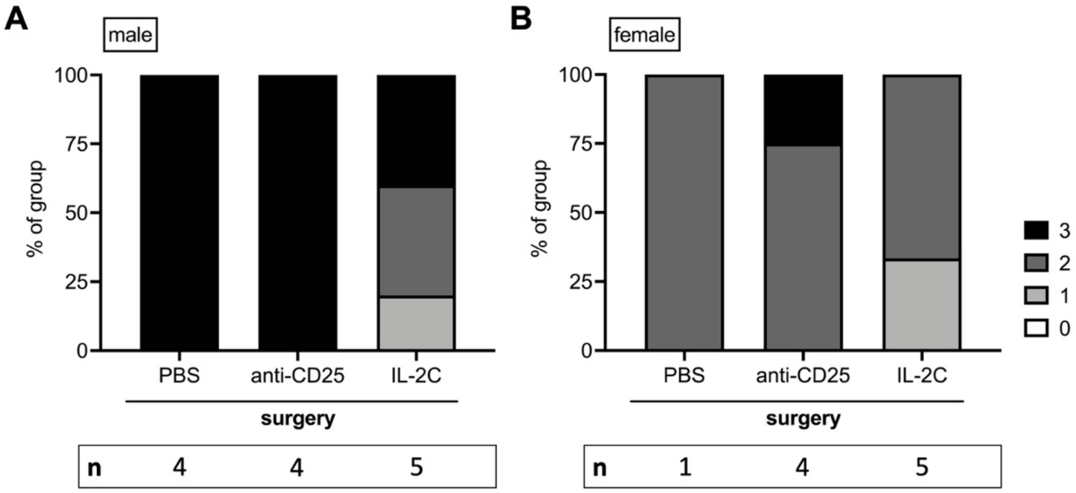

Supplementary Figure S11. Effect of preoperative regulatory T cell levels on postoperative atherosclerotic plaque complexity stratified by sex.
